# Prevalence of osteosarcopenia and associated factors among older Vietnamese outpatients: A cross-sectional study

**DOI:** 10.1371/journal.pone.0359850

**Published:** 2026-10-26

**Authors:** Long H. Ngo, Huong T. T. Hoang, Hoa T. Nguyen, An V. Tran, Van T. H. Nguyen

**Affiliations:** 1 Can Tho University of Medicine and Pharmacy, Can Tho City, Vietnam; 2 University of Medicine and Pharmacy, Hue University, Hue City, Vietnam; UBC: The University of British Columbia, CANADA

## Abstract

**Background:**

Osteosarcopenia – the concurrent presence of sarcopenia and low bone mass, including osteopenia or osteoporosis – is emerging as a significant health issue in aging populations. This study aimed to estimate the prevalence of osteosarcopenia and identify factors associated with prevalent osteosarcopenia among older Vietnamese outpatients.

**Materials and methods:**

A cross-sectional study was conducted on 389 outpatients aged ≥60 years at Can Tho University of Medicine and Pharmacy Hospital from 01/202511/2025. Osteosarcopenia was defined by coexistent low bone mass (osteopenia/osteoporosis via DXA) and sarcopenia defined according to AWGS 2019 as low muscle mass accompanied by low muscle strength, low physical performance or both. Logistic regression analyses were used to assess factors associated with osteosarcopenia. All data were analysed using R version 4.5.5.

**Results:**

The overall prevalence of osteosarcopenia was 26.7% (95% CI 22.6–31.3%). Prevalence was similar in women and men (26.3% vs 28.6%, p = 0.68). On multivariable analysis, older age (per 1-year increase; OR 1.06, 95% CI 1.02–1.10), lower BMI (per 1 kg/m^2^ increase; OR 0.57, 95% CI 0.50–0.65), coronary artery disease (OR 2.07, 95% CI 1.09–3.95), and diabetes (OR 2.48, 95% CI 1.35–4.56) were independently associated with osteosarcopenia.

**Conclusion:**

Osteosarcopenia affected about one in four older outpatients in this Vietnamese cohort. Older age, lower body mass index, coronary artery disease, and diabetes were independently associated with osteosarcopenia. These findings highlight the importance of early screening and integrated management of bone and muscle health in older adults.

## Introduction

Osteosarcopenia (OS) refers to the concurrent presence of sarcopenia and low bone mass, including osteopenia or osteoporosis in an individual, reflecting the interrelated degeneration of bone and muscle in old age [1]. In the context of population aging, osteosarcopenia has emerged as a growing public health concern worldwide [1]. Older adults with OS have been shown to face significantly elevated risks of adverse outcomes, including falls, fragility fractures, disability and mortality [2,3]. Globally, a recent meta-analysis estimated that roughly one in five older adults may have osteosarcopenia [3]. The likelihood of osteosarcopenia rises substantially with advancing age – approaching 50% in those over 75 years [1].

Multiple factors associated with osteosarcopenia have been identified in the international literature. Advancing age has consistently been associated with osteosarcopenia [3]. In addition, underweight older adults and those with malnutrition have higher odds of developing osteosarcopenia [4]. Moreover, chronic diseases such as type 2 diabetes mellitus and cardiovascular disease are linked to greater osteosarcopenia risk and insufficient physical activity further exacerbates age-related muscle and bone loss [4,5].

Despite growing global research, there is a notable lack of data on osteosarcopenia in Southeast Asia, including Vietnam. Most studies to date have been conducted in Western populations or East Asian countries, while very few have examined Southeast Asian cohorts [3]. In Vietnam, osteoporosis, with a reported prevalence of approximately 27% among older women and 13% among older men [6], has been extensively investigated in epidemiological research [6–8]. Sarcopenia has likewise been examined as an independent geriatric syndrome [9]. Nevertheless, evidence regarding the combined manifestation of these two conditions, conceptualized as osteosarcopenia, remains lacking. Therefore, the present study was undertaken to address this critical knowledge gap by comprehensively investigating osteosarcopenia in an older adult population in Vietnam, a rapidly aging middle-income country undergoing significant demographic and epidemiological transitions. Specifically, we aimed to estimate the overall prevalence of osteosarcopenia and its prevalence across selected comorbid conditions and to identify potential factors associated with osteosarcopenia.

## Methods

### Study setting and population

This cross-sectional study was conducted at the Outpatient Department, Can Tho University of Medicine and Pharmacy Hospital, Vietnam, from January 2025 to November 2025. All individuals aged ≥60 years who attended the clinic during the study period and provided written informed consent were considered eligible. Participants were excluded if they were unable to ambulate or perform physical activity; had hearing or visual impairment that precluded assessment; were unable to understand or respond to the questionnaire; were unable or unwilling to follow instructions for performance-based tests; had residual deficits from ischemic or hemorrhagic stroke; had a history of limb amputation for any cause; had current or prior treatment for osteoporosis, or a history of hypothyroidism or hypoparathyroidism; had severe acute illnesses (e.g., sepsis, acute respiratory failure, severe hepatic failure, severe renal failure, acute stroke, hyperglycemic coma, or hypoglycemia); or had end-stage chronic kidney disease. A total of 389 participants were included in the final analysis (Fig 1). The study protocol was reviewed and approved by the Institutional Ethics Committee of Hue University of Medicine and Pharmacy, Hue University, Vietnam (Approval number: H2024/535; approval date: August 19, 2024). All participants provided written informed consent before enrollment.

**Fig 1:**
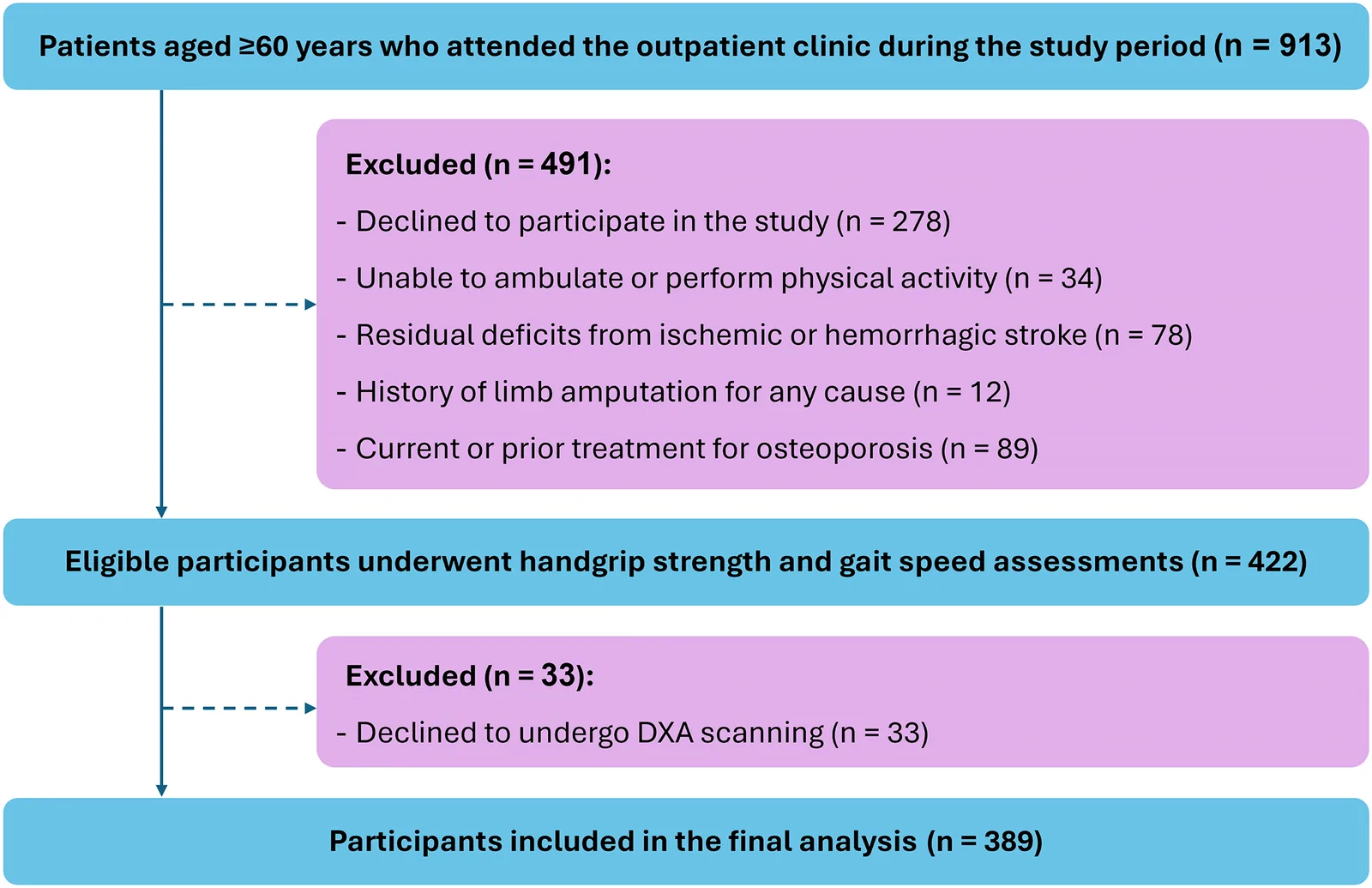
Flow diagram of participant recruitment and inclusion in the final analysis.

### Variables and measurements

#### Osteosarcopenia.

Osteosarcopenia (OS) was defined as the coexistence of sarcopenia and low bone mass (osteopenia) or osteoporosis [10]. Appendicular skeletal muscle mass (ASM, kg) and DXA-derived areal bone mineral density (BMD, g/cm^2^) at the lumbar spine (L1–L4), total hip and femoral neck were assessed using dual-energy X-ray absorptiometry (DXA) (PRODIGY/DPX HV-P8; GE Healthcare, USA). Hip BMD was measured on the non-dominant side. To determine leg dominance, participants were asked to stand on one leg; the leg not selected as the supporting leg was classified as the non-dominant leg. The dominant hip was assessed if a femoral head prosthesis or metallic implant was present on the non-dominant side. All scans were acquired by one trained DXA technologist and subsequently reviewed and analyzed by one investigator. The coefficients of variation were 2.0% for ASM, 1.8% for the lumbar spine and 1.5% for the proximal femur. The DXA system underwent routine quality control using a standard spine phantom with a known aBMD of 1.0 g/cm^2^. In our laboratory, phantom scans were performed twice daily, in the morning and afternoon. Manufacturer-provided Asian reference data were used for reporting because locally validated population-specific Vietnamese reference data are not routinely available for clinical DXA interpretation [11].

Low BMD was classified according to the World Health Organization (WHO) criteria based on T-scores: osteopenia was defined as a T-score between −1.0 and −2.5, and osteoporosis as a T-score ≤ −2.5 [12]. For classification, the lowest T-score among the lumbar spine (L1–L4), total hip and femoral neck was used.

Sarcopenia was defined according to the Asian Working Group for Sarcopenia 2019 (AWGS 2019) [13]. ASM (kg) was calculated as the sum of lean mass of both arms and both legs. The appendicular skeletal muscle mass index (ASMI) was calculated as ASM divided by height squared (kg/m^2^). Low ASMI was defined as <7.0 kg/m^2^ in men and <5.4 kg/m^2^ in women. Muscle strength was assessed by handgrip strength using a hydraulic hand dynamometer (Jamar™, USA) [13], which was regularly calibrated. Participants were seated with the elbow flexed at 90° and were instructed to squeeze gradually to maximum effort and maintain the contraction for 3 seconds. Grip strength was measured in both hands and the maximum value from either hand was used for analysis. Low muscle strength was defined as <28 kg in men and <18 kg in women [13]. Physical performance was evaluated by usual gait speed over a 6-m walk; low physical performance was defined as gait speed <1.0 m/s [13]. According to AWGS 2019, sarcopenia was diagnosed when low ASMI was accompanied by low muscle strength, low physical performance, or both. Severe sarcopenia was defined as the presence of low ASMI, low muscle strength and low physical performance. For the definition of osteosarcopenia, participants with either sarcopenia or severe sarcopenia were considered to have sarcopenia [13].

#### Participant characteristics, lifestyle factors and comorbidity ascertainment.

Participant characteristics and lifestyle factors included sex, age, measured height and weight, body mass index (BMI), living arrangement, smoking status, and alcohol use. Height and weight were measured by outpatient clinic nurses as part of the routine assessment upon the participant’s arrival. Participants who provided written informed consent underwent the remaining study assessments during the same outpatient visit. BMI was calculated as weight in kilograms divided by height in meters squared (kg/m^2^) and categorized according to Asian-specific BMI thresholds: normal (18.5–22.9 kg/m^2^), overweight (23.0–27.4 kg/m^2^) and obesity (≥27.5 kg/m^2^) [14]. Living arrangement was categorized as living with family or living alone; no participants resided in nursing homes. Lifestyle factors included current smoking (self-reported ongoing cigarette smoking at the time of assessment) and alcohol use. Alcohol consumption was defined as current drinking, with heavy alcohol use operationalized as ≥5 alcohol units/day or ≥15 units/week based on the study questionnaire. History of falls, fractures and family history of fracture were also recorded.

Comorbidities were ascertained from medical history, current medications and/or newly established diagnoses during the study enrollment visit. Hypertension was defined as systolic blood pressure ≥140 mmHg and/or diastolic blood pressure ≥90 mmHg and/or current use of antihypertensive medication [15]. Coronary artery disease (CAD) was defined as a prior history of acute coronary syndrome or the presence of chest pain accompanied by abnormalities on a routine 12-lead electrocardiogram [16]. Congestive heart failure (CHF) was defined by symptoms consistent with heart failure together with abnormal findings on echocardiography [17]. Diabetes mellitus was defined according to the American Diabetes Association criteria using any of the following: fasting plasma glucose ≥126 mg/dL (7.0 mmol/L), HbA1c ≥ 6.5% or random plasma glucose ≥200 mg/dL with classic hyperglycemic symptoms [18]. Chronic kidney disease was defined according to KDIGO as abnormalities of kidney structure or function present for ≥3 months with health implications [19]. Chronic liver disease in this study referred to cirrhosis only (no other chronic liver conditions were recorded).

### Data analysis

All analyses were conducted in R (R Foundation for Statistical Computing, Vienna, Austria) [20]. Data were cleaned and managed using the dplyr and tidyr packages. Participant characteristics were summarized using tableone: continuous variables are presented as mean ± standard deviation and compared between groups using the independent-samples t test; categorical variables are presented as n (%) and compared using the chi-square test, while exact methods were applied when appropriate (Fisher’s exact test or the Fisher–Freeman–Halton exact test, implemented via stats and DescTools). The overall prevalence of osteosarcopenia and its prevalence across comorbid conditions were calculated with 95% confidence intervals (CIs) for proportions (Wilson score method, via binom) and visualized using column charts with error bars (95% CIs) created with ggplot2. Associations between potential factors and prevalent osteosarcopenia were first examined using univariable logistic regression. Age and BMI were analyzed as continuous variables, with ORs reported per 1-year and per 1 kg/m^2^ increase, respectively. Variables with p < 0.20 in univariable analyses were considered for inclusion in the multivariable model [21]. To reduce the instability of adjusted estimates due to sparse data, congestive heart failure, stroke, and chronic liver disease were excluded from the primary conventional multivariable logistic regression model but were retained in the exploratory univariable analyses. These variables were subsequently included in a sensitivity analysis using Firth penalized multivariable logistic regression. Statistical significance was defined as a two-sided p < 0.05. Multicollinearity was assessed using variance inflation factors. The functional forms of age and BMI were examined using the Box–Tidwell approach, with a spline-based analysis performed as a sensitivity analysis. Overall model fit was assessed using the Hosmer–Lemeshow test, and potentially influential observations were evaluated using Cook’s distances. Results were reported as ORs with 95% CIs; profile-likelihood 95% CIs were used for the Firth model, implemented using the logistf package. Adjusted ORs from the primary model were displayed in a forest plot using ggplot2.

## Results

Among the 389 participants, the mean age was 70.3 ± 7.3 years (range, 60–96 years) and 312 (80.2%) were women. The overall prevalence of osteosarcopenia was 26.7% (95% CI 22.6%–31.3%). Detailed sex-stratified age and musculoskeletal characteristics are provided in S1 Table.

Compared with participants without osteosarcopenia, those with osteosarcopenia were older and had lower BMI. The prevalence of hypertension, congestive heart failure, and diabetes was significantly higher in the osteosarcopenia group, whereas sex distribution, living arrangement, smoking and alcohol use, fall and fracture history, family history of fracture, glucocorticoid use, coronary artery disease, stroke, dyslipidemia, chronic kidney disease and chronic liver disease did not differ significantly between groups. Bone mineral density classification differed substantially, with a predominance of osteoporosis in the osteosarcopenia group and sarcopenia was present in all individuals with osteosarcopenia by definition (Table 1).

**Table 1:** Participant characteristics according to osteosarcopenia status.

Characteristics	OS (n = 104)	Non-OS (n = 285)	Total (n = 389)	p
Male, n (%)	22 (21.2)	55 (19.3)	77 (19.8)	0.684^a^
Age (years), mean (SD)	72.0 (6.9)	69.7 (7.3)	70.3 (7.3)	**0.004** ^**b**^
Weight (kg)	48.7 (6.6)	58.7 (8.5)	56.0 (9.2)	**<0.001** ^**b**^
Height (m)	1.5 (0.1)	1.5 (0.1)	1.5 (0.1)	0.485^b^
BMI (kg/m^2^), mean (SD)	20.6 (2.6)	24.6 (3.2)	23.5 (3.5)	**<0.001** ^**b**^
BMI Classification, n (%)				**<0.001** ^**c**^
Underweight	22 (21.2)	4 (1.4)	26 (6.7)	
Normal weight	64 (61.5)	89 (31.2)	153 (39.3)	
Overweight	14 (13.5)	81 (28.4)	95 (24.4)	
Obesity	4 (3.8)	111 (38.9)	115 (29.6)	
Living arrangement, n (%)				0.242^a^
With family	89 (85.6)	256 (89.8)	345 (88.7)	
Alone	15 (14.4)	29 (10.2)	44 (11.3)	
Smoking history, n (%)	14 (13.5)	33 (11.6)	47 (12.1)	0.614^a^
Alcohol use, n (%)	11 (10.6)	27 (9.5)	38 (9.8)	0.746^a^
Fall history, n (%)	22 (21.2)	53 (18.6)	75 (19.3)	0.571^a^
Fracture history, n (%)	16 (15.4)	25 (8.8)	41 (10.5)	0.060^a^
Family history of fracture, n (%)	6 (5.8)	9 (3.2)	15 (3.9)	0.236^a^
Glucocorticoids use, n (%)	32 (30.8)	79 (27.7)	111 (28.5)	0.555^a^
Hypertension, n (%)	58 (55.8)	120 (42.1)	178 (45.8)	**0.017** ^**a**^
CAD, n (%)	33 (31.7)	63 (22.1)	96 (24.7)	0.051^a^
CHF, n (%)	4 (3.8)	2 (0.7)	6 (1.5)	**0.040** ^**d**^
Stroke, n (%)	3 (2.9)	2 (0.7)	5 (1.3)	0.121^d^
Diabetes, n (%)	45 (43.3)	72 (25.3)	117 (30.1)	**<0.001** ^**a**^
Dyslipidemia, n (%)	36 (34.6)	87 (30.5)	123 (31.6)	0.443^a^
Chronic kidney disease, n (%)	8 (7.7)	15 (5.3)	23 (5.9)	0.369^a^
Chronic liver disease, n (%)	2 (1.9)	8 (2.8)	10 (2.6)	1.000^a^
BMD classification, n (%)				**<0.001** ^**c**^
T-score ≥ −1.0	0 (0.0)	84 (29.5)	84 (21.6)	
−2.5 < T-score <−1.0	58 (55.8)	76 (26.7)	134 (34.4)	
T-score ≤ −2.5	46 (44.2)	125 (43.9)	171 (44.0)	
Sarcopenia, n (%)	104 (100)	28 (9.8)	132 (33.9)	**<0.001** ^**c**^

^a^Chi-square test, ^b^Independent Sample T test, ^c^Fisher Freeman Halton Exact test, ^d^Fisher’s Exact test.

Fig 2 shows that the prevalence of osteosarcopenia was highest among participants with sarcopenia (78.8%, 95% CI 71.1–84.9), followed by those with congestive heart failure (66.7%, 30.0–90.3) and prior stroke (60.0%, 23.1–88.2). In contrast, the lowest prevalence was observed in chronic liver disease (20.0%, 5.7–51.0). Across most cardiometabolic conditions, including hypertension, coronary artery disease, diabetes and dyslipidemia, osteosarcopenia prevalence was consistently around one-third.

**Fig 2:**
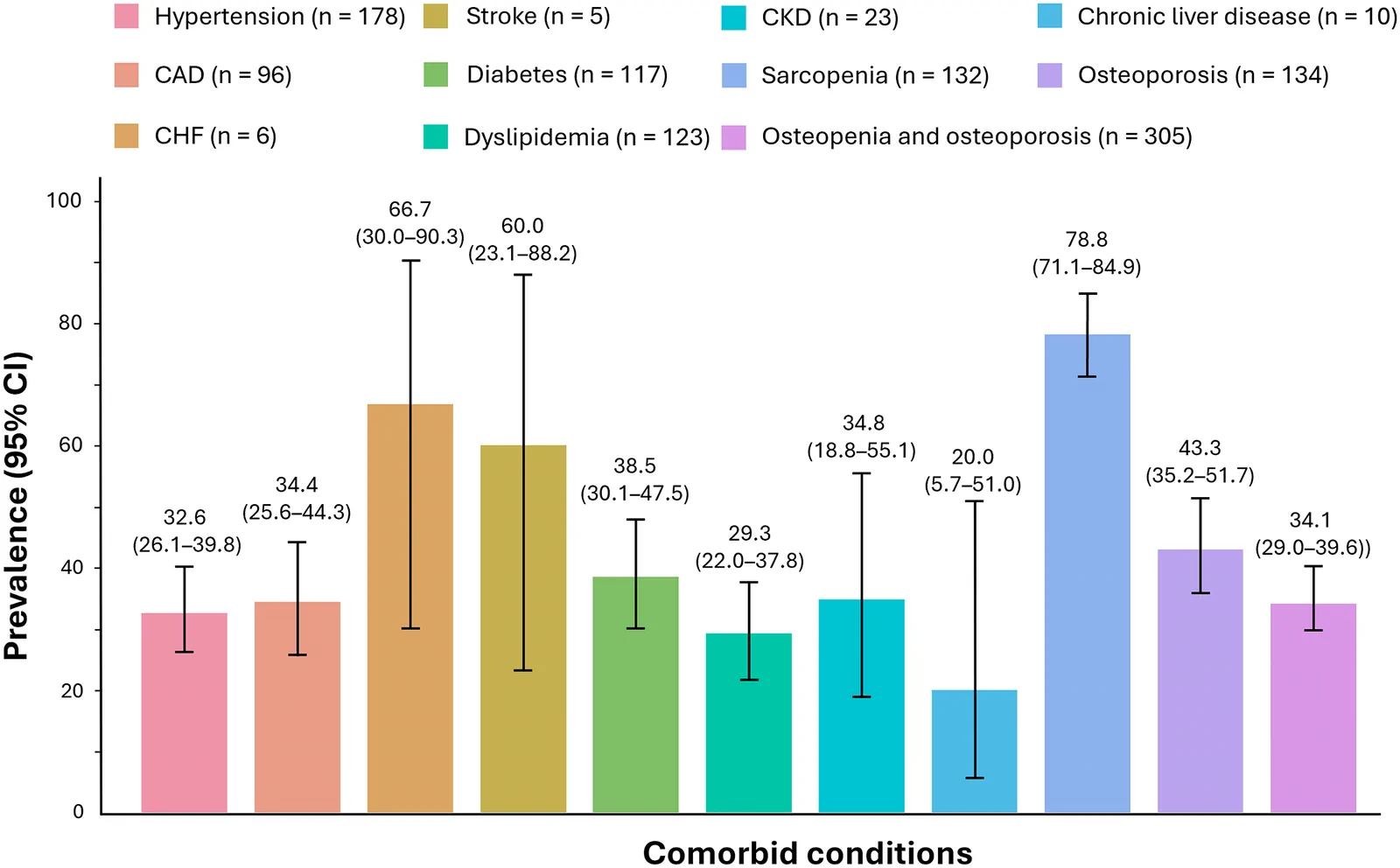
Prevalence of osteosarcopenia across comorbid conditions. Columns represent prevalence estimates, and error bars represent 95% confidence intervals calculated using the Wilson score method.

In univariable analyses, older age, hypertension, congestive heart failure, and diabetes were significantly associated with higher odds of osteosarcopenia, whereas BMI showed an inverse association. The estimate for congestive heart failure should be interpreted cautiously because only six participants had this condition (Table 2).

**Table 2:** Univariable logistic regression analysis of factors associated with osteosarcopenia.

Factors	Unit of comparison	OR (95% CI)	p
Male	Yes	1.12 (0.63–1.93)	0.684
Age	+1 year	1.04 (1.01–1.08)	**0.005**
BMI	+1 kg/m^2^	0.59 (0.52–0.67)	**<0.001**
Smoking history	Yes	1.19 (0.59–2.28)	0.614
Alcohol use	Yes	1.13 (0.52–2.31)	0.746
Fall history	Yes	1.17 (0.67–2.05)	0.572
Fracture history	Yes	1.89 (0.97–3.70)	0.063
Family history of fracture	Yes	1.88 (0.65–5.41)	0.243
Glucocorticoids use	Yes	1.16 (0.70–1.88)	0.555
Hypertension	Yes	1.73 (1.10–2.74)	**0.017**
CAD	Yes	1.64 (0.99–2.69)	0.051
Chronic heart failure	Yes	5.66 (1.09–41.28)	**0.047**
Stroke	Yes	4.20 (0.69–32.25)	0.119
Diabetes	Yes	2.26 (1.41–3.62)	**<0.001**
Dyslipidemia	Yes	1.20 (0.74–1.93)	0.443
Chronic kidney disease	Yes	1.50 (0.59–3.57)	0.369
Chronic liver disease	Yes	0.68 (0.10–2.76)	0.626

In multivariable analysis (Fig 3), older age (per 1-year increase; OR 1.06, 95% CI 1.02–1.10), coronary artery disease (OR 2.07, 95% CI 1.09–3.95) and diabetes (OR 2.48, 95% CI 1.35–4.56) remained independently associated with osteosarcopenia. BMI remained inversely associated with osteosarcopenia (per 1 kg/m^2^ increase; OR 0.57, 95% CI 0.50–0.65), indicating that each 1 kg/m^2^ increase in BMI was associated with lower odds of osteosarcopenia. Hypertension was not independently associated with osteosarcopenia in the primary multivariable model. Results of the Firth penalized sensitivity analysis including comorbidities with sparse cell counts are presented in S2 Table.

**Fig 3:**
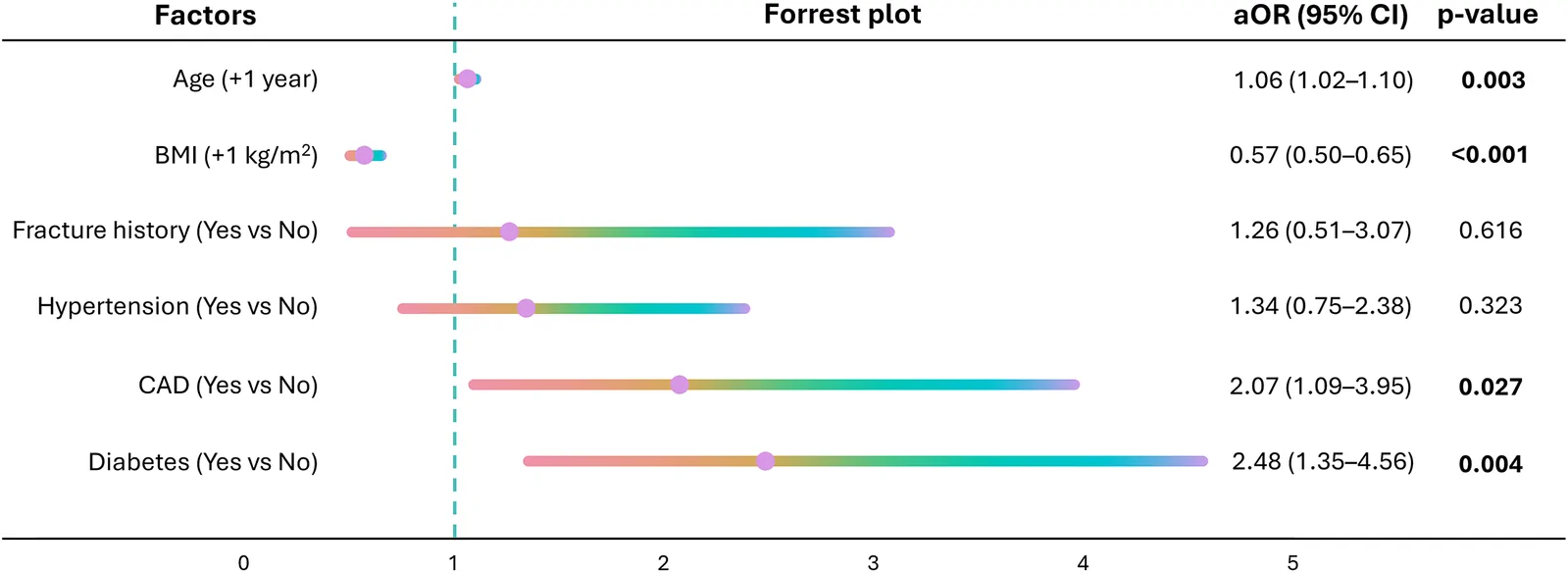
Multivariable logistic regression analysis of factors associated with osteosarcopenia. Adjusted odds ratios and 95% confidence intervals are shown for the primary model (n = 389; 104 participants with osteosarcopenia and 285 without osteosarcopenia). The reference category for fracture history, hypertension, coronary artery disease, and diabetes was the absence of the respective condition. Age and BMI were modeled per 1-year and per 1 kg/m^2^ increase, respectively. Points represent adjusted odds ratios, horizontal lines represent 95% confidence intervals, and the vertical dashed line at 1.0 indicates no association. Estimates shown in bold indicate p < 0.05.

## Discussion

In this cross-sectional analysis of older Vietnamese outpatients, osteosarcopenia was identified in more than one-quarter of the cohort (26.7%). The distribution was similar between sexes. Advanced age, lower body mass index, coronary artery disease and diabetes mellitus were independently associated with osteosarcopenia. Collectively, these results underscore the substantial clinical relevance of osteosarcopenia in this population and indicate a strong association with advancing age, reduced adiposity and cardiometabolic comorbidities.

The prevalence of osteosarcopenia (26.7%) observed in our cohort lies within the wide range reported globally. A recent meta-analysis estimated a pooled prevalence of approximately 21% among older adults, situating our findings slightly above the overall average [3]. This difference may be attributable to the hospital-based nature of our sample, which likely encompassed individuals with a higher burden of comorbid conditions. In contrast to many prior studies documenting a predominance of osteosarcopenia among women [5], we identified comparable prevalence between sexes. Although osteoporosis is generally more common in women [22], age-related decline in skeletal muscle mass and function among men may attenuate sex disparities in the combined phenotype of bone and muscle loss [23]. Furthermore, the lack of a significant difference in osteosarcopenia prevalence between men and women in our cohort may be attributable to the relatively small number of male participants, which could have limited statistical power, or to specific demographic and clinical features unique to our study population.

Consistent with prior research, increasing age was strongly associated with osteosarcopenia [1]. The co-occurrence of osteoporosis and sarcopenia in osteosarcopenia is believed to stem from convergent age-related mechanisms. With advancing age, immune and endocrine dysregulation (immunosenescence, sex hormone deficiencies, secondary hyperparathyroidism, etc.) lead to simultaneous bone and muscle catabolism [24,25]. There is also direct biochemical crosstalk between musculoskeletal tissues, whereby muscle-derived myokines contribute to the regulation of bone formation and bone-derived osteokines modulate muscle metabolism [26]. In osteosarcopenia, these signaling pathways become impaired. Additionally, fatty infiltration of muscle and bone marrow increases with age and inactivity, causing local lipotoxic effects that further weaken both tissues [27]. This explains why poor nutrition and sedentarism are major risk factors for osteosarcopenia in the elderly [10]. The inverse association of low BMI with osteosarcopenia in our study likely reflects the impact of chronic undernutrition and frailty – insufficient protein/calorie intake leads to loss of both bone density and muscle mass. Conversely, higher BMI may reflect greater mechanical loading and nutritional reserve, which could partly explain the observed inverse association between BMI and osteosarcopenia. However, BMI alone does not distinguish lean mass from fat mass, and its association with osteosarcopenia may vary according to body composition [3,28]. Moreover, the independent associations of diabetes and coronary artery disease with osteosarcopenia observed in our study are consistent with evidence linking chronic cardiometabolic conditions to concurrent bone and muscle loss [29]. For instance, Moretti et al. (2022) reported that post-menopausal women with type 2 diabetes had about five times higher odds of osteosarcopenia than non-diabetics [30]. Patients with long-standing diabetes often exhibit both reduced bone strength and accelerated muscle wasting due to hyperglycemia-induced tissue damage and hormonal resistance [30]. Similarly, atherosclerotic cardiovascular disease contributes to a chronic pro-inflammatory milieu and reduced physical activity, both of which may accelerate musculoskeletal deterioration. These pathophysiological considerations support the concept that osteosarcopenia arises from interconnected biological mechanisms rather than the simple coexistence of two independent disorders. In our analysis, hypertension and heart failure showed significant associations with osteosarcopenia in univariable models; however, these relationships were no longer evident after multivariable adjustment, suggesting that the unadjusted associations may have been confounded by age and other covariates. Taken together, our findings reinforce the multifactorial pathogenesis of osteosarcopenia described in previous studies, while also suggesting potential population-specific patterns within our cohort.

Our findings further emphasize that osteosarcopenia constitutes a clinically significant geriatric syndrome that requires early recognition and integrated management strategies. Older individuals presenting with concomitant low bone mass and reduced muscle quantity or function represent a particularly vulnerable subgroup. Evidence from international cohorts indicates that patients with osteosarcopenia face markedly increased risks of falls, fragility fractures and premature mortality compared with those affected by either osteoporosis or sarcopenia alone [1]. From a clinical perspective, the coexistence of bone fragility and poor muscle function greatly increases the risk of serious events such as hip fractures, which are linked to high mortality, long-term disability and substantial healthcare costs. Therefore, clinicians should remain alert to the possibility of osteosarcopenia in older adults, particularly those with chronic diseases, frailty, or declining physical performance. Simple screening tools, such as handgrip strength or usual gait speed to evaluate muscle function, together with bone mineral density measurement by dual-energy X-ray absorptiometry, can be readily applied in primary care and geriatric settings to support early detection of this high-risk condition [1,31,32]. Early recognition enables timely intervention – optimal management of osteosarcopenia requires addressing both its components. Guidelines emphasize treating the bone aspect with appropriate osteoporosis medications (calcium/vitamin D supplementation and antiresorptive or anabolic therapies) while simultaneously implementing interventions for muscle strengthening, such as protein supplementation and progressive resistance exercise training [33–36]. Multidisciplinary measures including fall prevention strategies and physical therapy are also essential. In the context of Vietnam, increasing awareness of osteosarcopenia among healthcare providers is crucial so that older adults with associated clinical conditions, such as diabetes or coronary artery disease, receive integrated bone–muscle health assessments. Such an approach has the potential to reduce fractures, preserve independence and improve overall quality of life in our aging population. By generating context-specific data from Southeast Asia, a region that remains underrepresented in the current literature, this study not only provides essential evidence to inform clinical practice and public health strategies at the national level, but also contributes to the global understanding of osteosarcopenia across diverse ethnic and socioeconomic settings, thereby supporting the development of more inclusive and regionally adaptable approaches to healthy aging.

This study contributes additional data on osteosarcopenia in Vietnam and, to our knowledge, is among the first to examine its prevalence and associated factors in a Vietnamese population. A key strength is the application of established diagnostic criteria, including AWGS 2019 for sarcopenia and WHO definitions for low bone mass, together with objective DXA measurements to enhance the accuracy of case classification. Nevertheless, several limitations should be acknowledged. First, the cross-sectional design precludes causal inference and the observed associations cannot clarify temporal relationships. For instance, it remains uncertain whether specific comorbidities contribute to the development of osteosarcopenia or whether underlying osteosarcopenia predisposes individuals to these conditions. Second, the study population was recruited from a single-center outpatient clinic and may not fully represent the broader elderly population. Older adults seeking hospital-based care may differ systematically in health status and comorbidity burden from community-dwelling peers. Thus, the observed prevalence should be interpreted as reflecting older hospital outpatients rather than the general elderly population. In addition, the exclusion of individuals with advanced diseases, such as end-stage renal disease or recent stroke, as well as those receiving osteoporosis treatment, was intended to reduce confounding but may have led to an underestimation of prevalence by omitting more frail or previously treated patients. Third, the predominance of women in the cohort (approximately 80%) and the relatively limited number of male participants may have reduced statistical power to detect sex-specific differences. This unbalanced sex distribution may also have influenced the overall prevalence estimate and should be considered when interpreting the study findings. Furthermore, we did not directly assess nutritional status beyond BMI, dietary intake including calcium intake, vitamin D status, physical activity using a validated questionnaire, or inflammatory biomarkers. Assessment of these factors could provide further insight into the mechanisms underlying osteosarcopenia. Finally, given the hospital-based design, caution is warranted when extrapolating these findings to other clinical settings or ethnic populations. Despite these constraints, our study provides foundational data on osteosarcopenia in Vietnam and underscores priorities for future research.

## Conclusion

Osteosarcopenia was a common condition among older Vietnamese adults visiting a hospital outpatient clinic, affecting approximately one in four participants. Advanced age, low body mass index, coronary artery disease and type 2 diabetes mellitus were identified as independent factors associated with osteosarcopenia. These findings support consideration of routine screening for combined bone and muscle loss among older hospital outpatients, particularly those with the associated characteristics identified in this study, together with integrated approaches to bone and muscle health.

## Supporting information

S1 Table Age and musculoskeletal characteristics of the study participants. (DOCX)

S2 Table Sensitivity analysis using Firth penalized multivariable logistic regression including comorbidities with sparse cell counts. (DOCX)

S1 Checklist STROBE-checklist for cross-sectional studies. (DOCX)

S1 Data De-identified raw data underlying the findings. (XLSX)
