# Supplementary material for: Prevalence of osteosarcopenia and associated factors among older Vietnamese outpatients: A cross-sectional study
Source: PLoS One. 2026 Oct 26;21(10):e0359850. doi: 10.1371/journal.pone.0359850 (PMC13704813; doi:10.1371/journal.pone.0359850)
Supplement: Supplementary file 1 [file pone.0359850.s006.pdf]

PLOS One

Osteosarcopenia in Elderly Vietnamese Adult: Prevalence and Risk factors

--Manuscript Draft--

**Manuscript Number:**	PONE-D-26-13624
**Article Type:**	Research Article
**Full Title:**	Osteosarcopenia in Elderly Vietnamese Adult: Prevalence and Risk factors
**Short Title:**	Osteosarcopenia in Elderly Vietnamese Adult: Prevalence and Risk factors
**Corresponding Author:**	Hoang Thanh Van Nguyen<br>Hue University of Medicine and Pharmacy<br>Hue, VIET NAM
**Keywords:**	osteosarcopenia; Prevalence; Risk factors; elderly patients; Vietnamese cohort
**Abstract:**	Background: Osteosarcopenia – the concurrent presence of osteoporosis and sarcopenia – is emerging as a significant health issue in aging populations. This study aimed to determine the prevalence and risk factors of osteosarcopenia in older Vietnamese outpatients.<br>Materials and methods: A cross-sectional study was conducted on 389 outpatients aged ≥60 years at Can Tho University of Medicine and Pharmacy Hospital from 01/2025 to 11/2025. Osteosarcopenia was defined by coexistent low bone mass (osteopenia/osteoporosis via DXA) and sarcopenia (low muscle strength and/or low muscle mass per AWGS 2019 criteria). Logistic regression analyses were used to assess factors associated with osteosarcopenia. All data were analysed using R enviroment 4.5.5 version.<br>Results: The overall prevalence of osteosarcopenia was 26.7% (95% CI 22.6–31.3%). Prevalence was similar in women and men (26.3% vs 28.6%, p=0.68). On multivariable analysis, older age (per +10 years; OR 1.77, 95% CI 1.20–2.65; p = 0.004), higher BMI (per +1 kg/m²; OR 0.57, 95% CI 0.49–0.64; p < 0.001), coronary artery disease (OR 2.12, 95% CI 1.11–4.09; p = 0.024), and diabetes (OR 2.60, 95% CI 1.41–4.86; p = 0.002) remained independently associated with osteosarcopenia.<br>Conclusion: Osteosarcopenia affected about one in four older outpatients in this Vietnamese cohort. Advanced age, low body mass index and chronic cardiometabolic conditions like coronary artery disease and diabetes were significant associated factors. These findings highlight the importance of early screening and integrated management of bone and muscle health in older adults.
**Order of Authors:**	Hoang Long Ngo
	Hoang Thanh Van Nguyen, PhD
	Viet An Tran, PhD
	Hoa Thai Nguyen, PhD
	Thi Thu Huong Hoang, PhD
**Additional Information:**	
**Question**	**Response**
**Financial Disclosure**<br><br>Enter a financial disclosure statement that describes the sources of funding for the work included in this submission. Review the submission guidelines for detailed requirements. View published research articles from *PLOS ONE* for specific examples.	The author(s) received no specific funding for this work.

Powered by Editorial Manager® and ProduXion Manager® from Aries Systems Corporation

This statement is required for submission and **will appear in the published article** if the submission is accepted. Please make sure it is accurate.<br><br>**Funded studies**<br>Enter a statement with the following details:<br>• Initials of the authors who received each award<br>• Grant numbers awarded to each author<br>• The full name of each funder<br>• URL of each funder website<br>• Did the sponsors or funders play any role in the study design, data collection and analysis, decision to publish, or preparation of the manuscript?<br><br>Did you receive funding for this work?	
**Competing Interests**<br><br>Use the instructions below to enter a competing interest statement for this submission. On behalf of all authors, disclose any competing interests that could be perceived to bias this work—acknowledging all financial support and any other relevant financial or non-financial competing interests.<br><br>This statement is required for submission and **will appear in the published article** if the submission is accepted. Please make sure it is accurate and that any funding sources listed in your Funding Information later in the submission form are also declared in your Financial Disclosure statement.<br><br>View published research articles from *PLOS ONE* for specific examples.	The authors have declared that no competing interests exist.

Powered by Editorial Manager® and ProduXion Manager® from Aries Systems Corporation

**NO authors have competing interests**<br><br>Enter: *The authors have declared that no competing interests exist.*<br><br>**Authors with competing interests**<br><br>Enter competing interest details beginning with this statement:<br><br>*I have read the journal's policy and the authors of this manuscript have the following competing interests: [insert competing interests here]*<br><br>* typeset	
**Ethics Statement**<br><br>Enter an ethics statement for this submission. This statement is required if the study involved:<br><br>• Human participants<br>• Human specimens or tissue<br>• Vertebrate animals or cephalopods<br>• Vertebrate embryos or tissues<br>• Field research<br><br>Write "N/A" if the submission does not require an ethics statement.<br><br>General guidance is provided below. Consult the submission guidelines for detailed instructions. **Make sure that all information entered here is included in the Methods section of the manuscript.**	This study was approved by the Institutional Ethics Committee of Hue University of Medicine and Pharmacy (Approval No: H2024/535). Written informed consent was obtained from all participants.

Powered by Editorial Manager® and ProduXion Manager® from Aries Systems Corporation

**Format for specific study types**<br><br>**Human Subject Research (involving human participants and/or tissue)**<br>• Give the name of the institutional review board or ethics committee that approved the study<br>• Include the approval number and/or a statement indicating approval of this research<br>• Indicate the form of consent obtained (written/oral) or the reason that consent was not obtained (e.g. the data were analyzed anonymously)<br><br>**Animal Research (involving vertebrate animals, embryos or tissues)**<br>• Provide the name of the Institutional Animal Care and Use Committee (IACUC) or other relevant ethics board that reviewed the study protocol, and indicate whether they approved this research or granted a formal waiver of ethical approval<br>• Include an approval number if one was obtained<br>• If the study involved *non-human primates*, add *additional details* about animal welfare and steps taken to ameliorate suffering<br>• If anesthesia, euthanasia, or any kind of animal sacrifice is part of the study, include briefly which substances and/or methods were applied<br><br>**Field Research**<br><br>Include the following details if this study involves the collection of plant, animal, or other materials from a natural setting:<br>• Field permit number<br>• Name of the institution or relevant body that granted permission	
**Data Availability**<br><br>Authors are required to make all data underlying the findings described fully available, without restriction, and from the time of publication. PLOS allows rare exceptions to address legal and ethical concerns. See the PLOS Data Policy and FAQ for detailed information.	Yes - all data are fully available without restriction

A Data Availability Statement describing where the data can be found is required at submission. Your answers to this question constitute the Data Availability Statement and **will be published in the article**, if accepted.<br><br>**Important:** Stating 'data available on request from the author' is not sufficient. If your data are only available upon request, select 'No' for the first question and explain your exceptional situation in the text box.<br><br>Do the authors confirm that all data underlying the findings described in their manuscript are fully available without restriction?	
**Describe where the data may be found in full sentences. If you are copying our sample text, replace any instances of XXX with the appropriate details.**<br><br>• If the data are **held or will be held in a public repository**, include URLs, accession numbers or DOIs. If this information will only be available after acceptance, indicate this by ticking the box below. For example: *All XXX files are available from the XXX database (accession number(s) XXX, XXX.)*.<br>• If the data are all contained **within the manuscript and/or Supporting Information files**, enter the following: *All relevant data are within the manuscript and its Supporting Information files.*<br>• If neither of these applies but you are able to provide **details of access elsewhere**, with or without limitations, please do so. For example:<br><br>*Data cannot be shared publicly because of [XXX]. Data are available from the XXX Institutional Data Access / Ethics Committee (contact via XXX) for researchers who meet the criteria for access to confidential data.*<br><br>*The data underlying the results presented in the study are available from (include the name of the third party*	All relevant data are within the manuscript and its Supporting Information files.

and contact information or URL).<br>• This text is appropriate if the data are owned by a third party and authors do not have permission to share the data.<br><br>* typeset	
Additional data availability information:	

Manuscript

Click here to access/download;Manuscript;1. Main manuscript-26Mar.docx

Osteosarcopenia in Elderly Vietnamese Adult: Prevalence and Risk factors

Category: Original Article

Long H. Ngo[1,2], Huong T. T. Hoang[2], Hoa T. Nguyen[1], An V. Tran[1], Van T. H. Nguyen[2]

Institution Affiliation:

[1]Can Tho University of Medicine and Pharmacy, Can Tho City, Vietnam;

[2]University of Medicine and Pharmacy, Hue University, Hue City, Vietnam;

Corresponding author

Dr. Van T. H. Nguyen

Email: hntvan@hueuni.edu.vn; nhtvan@huemed-univ.edu.vn

Abstract

Background: Osteosarcopenia – the concurrent presence of osteoporosis and sarcopenia – is emerging as a significant health issue in aging populations. This study aimed to determine the prevalence and risk factors of osteosarcopenia in older Vietnamese outpatients.

Materials and methods: A cross-sectional study was conducted on 389 outpatients aged ≥60 years at Can Tho University of Medicine and Pharmacy Hospital from 01/2025 to 11/2025. Osteosarcopenia was defined by coexistent low bone mass (osteopenia/osteoporosis via DXA) and sarcopenia (low muscle strength and/or low muscle mass per AWGS 2019 criteria). Logistic regression analyses were used to assess factors associated with osteosarcopenia. All data were analysed using R enviroment 4.5.5 version.

Results: The overall prevalence of osteosarcopenia was 26.7% (95% CI 22.6–31.3%). Prevalence was similar in women and men (26.3% vs 28.6%, p=0.68). On multivariable analysis, older age (per +10 years; OR 1.77, 95% CI 1.20–2.65; p = 0.004), higher BMI (per +1 kg/m²; OR 0.57, 95% CI 0.49–0.64; p < 0.001), coronary artery disease (OR 2.12, 95% CI 1.11–4.09; p = 0.024), and diabetes (OR 2.60, 95% CI 1.41–4.86; p = 0.002) remained independently associated with osteosarcopenia.

Conclusion: Osteosarcopenia affected about one in four older outpatients in this Vietnamese cohort. Advanced age, low body mass index and chronic cardiometabolic conditions like coronary artery disease and diabetes were significant associated factors. These findings highlight the importance of early screening and integrated management of bone and muscle health in older adults.

Keywords: osteosarcopenia (OS), prevalence, risk factors, elderly patients, Vietnamese cohort.

Introduction

Osteosarcopenia (OS) refers to the concurrent presence of osteoporosis and sarcopenia in an individual, reflecting the interrelated degeneration of bone and muscle in old age. In the context of population aging, osteosarcopenia has emerged as a growing public health concern worldwide.[1] Older adults with OS have been shown to face significantly elevated risks of adverse outcomes, including falls, fragility fractures, disability, and mortality.[2, 3] Globally, a recent meta-analysis estimated that roughly one in five older adults may have osteosarcopenia.[3] The likelihood of OS rises substantially with advancing age – approaching 50% in those over 75 years.[1]

Multiple risk factors for osteosarcopenia have been identified in the international literature. Advancing age is a well-established non-modifiable risk factor.[3] In addition, underweight older adults and those with malnutrition have higher odds of developing osteosarcopenia.[4] Moreover, chronic diseases such as type 2 diabetes mellitus and cardiovascular disease are linked to greater osteosarcopenia risk, and

insufficient physical activity further exacerbates age-related muscle and bone loss.[4, 5]

Despite growing global research, there is a notable lack of data on osteosarcopenia in Southeast Asia, including Vietnam. Most studies to date have been conducted in Western populations or East Asian countries, while very few have examined Southeast Asian cohorts. In Vietnam, osteoporosis, with a reported prevalence of approximately 27% among older women and 13% among older men,[6] has been extensively investigated in epidemiological research. Sarcopenia has likewise been examined as an independent geriatric syndrome.[7] Nevertheless, evidence regarding the combined manifestation of these two conditions, conceptualized as osteosarcopenia, remains lacking. Therefore, the present study was undertaken to address this critical knowledge gap by comprehensively investigating osteosarcopenia in an older adult population in Vietnam, a rapidly aging middle-income country undergoing significant demographic and epidemiological transitions. Specifically, we aimed to estimate the prevalence of osteosarcopenia and to identify its associated risk factors using standardized diagnostic

criteria. By generating context-specific data from Southeast Asia, a region that remains underrepresented in the current literature, this study not only provides essential evidence to inform clinical practice and public health strategies at the national level, but also contributes to the global understanding of osteosarcopenia across diverse ethnic and socioeconomic settings, thereby supporting the development of more inclusive and regionally adaptable approaches to healthy aging.

Methods

Study setting and population

This cross-sectional study was conducted at the Outpatient Department, Can Tho University of Medicine and Pharmacy Hospital, Viet Nam, from January 2025 to November 2025. All individuals aged ≥60 years who attended the clinic during the study period and provided written informed consent were considered eligible. Participants were excluded if they were unable to ambulate or perform physical activity; had hearing or visual impairment that precluded assessment; were unable to understand or respond to the questionnaire; were unable or unwilling to

follow instructions for performance-based tests; had residual deficits from ischemic or hemorrhagic stroke; had a history of limb amputation for any cause; had current or prior treatment for osteoporosis, or a history of hypothyroidism or hypoparathyroidism; had severe acute illnesses (e.g., sepsis, acute respiratory failure, severe hepatic failure, severe renal failure, acute stroke, hyperglycemic coma, or hypoglycemia); or had end-stage chronic kidney disease. A total of 389 participants were included in the final analysis.

Variables and measurements

Osteosarcopenia

Osteosarcopenia (OS) was defined as the coexistence of sarcopenia and low bone mass (osteopenia) or osteoporosis.[8] Appendicular skeletal muscle mass (ASM) and bone mineral density (BMD) at the lumbar spine (L1–L4), total hip, and femoral neck were assessed using dual-energy X-ray absorptiometry (DXA) (PRODIGY/DPX HV-P8; GE Healthcare, USA). The coefficients of variation were 1.8% for the lumbar spine and 1.5% for the proximal femur. The DXA system underwent routine quality control by scanning a standard spine phantom with a known areal BMD

(1.0 g/cm²) before clinical use; in our laboratory, phantom scans were performed twice daily (morning and afternoon). It is recommended that a spine phantom be scanned daily (or at least three times weekly) with review of the phantom trend data. Asian reference data provided by the manufacturer were used for reporting, as locally validated population-specific Vietnamese reference data are not routinely available for clinical DXA interpretation.[9]

Low BMD was classified according to the World Health Organization (WHO) criteria based on T-scores: osteopenia was defined as a T-score between −1.0 and −2.5, and osteoporosis as a T-score ≤ −2.5.[10] For classification, the lowest T-score among the lumbar spine (L1–L4), total hip, and femoral neck was used.

Sarcopenia was defined according to the Asian Working Group for Sarcopenia 2019 (AWGS 2019).[11] ASM (kg) was calculated as the sum of lean mass of both arms and both legs. The appendicular skeletal muscle mass index (ASMI) was calculated as ASM divided by height squared (kg/m^2). Low ASMI was defined as <7.0 kg/m^2 in men and <5.4 kg/m^2 in women. Muscle strength was assessed by handgrip strength using a

hydraulic hand dynamometer (Jamar™, USA),[11] which was regularly calibrated. Participants were seated with the elbow flexed at 90°, and were instructed to squeeze gradually to maximum effort and maintain the contraction for 3 seconds. Grip strength was measured in both hands, and the maximum value from either hand was used for analysis. Low muscle strength was defined as <28 kg in men and <18 kg in women.[11] Physical performance was evaluated by usual gait speed over a 6-m walk; low physical performance was defined as gait speed <1.0 m/s.[11]

Baseline characteristics, lifestyle factors, and comorbidity ascertainment

Baseline characteristics included sex, age, body mass index (BMI), and living arrangement. BMI was calculated as weight in kilograms divided by height in meters squared (kg/m^2) and categorized according to Asian-specific BMI thresholds: normal (18.5–22.9 kg/m^2), overweight (23.0–27.4 kg/m^2), and obesity (≥27.5 kg/m^2).[12] Living arrangement was categorized as living with family or living alone; no participants resided in nursing homes. Lifestyle factors included current smoking (self-reported ongoing cigarette smoking at the time of assessment) and

alcohol use. Alcohol consumption was defined as current drinking, with heavy alcohol use operationalized as ≥5 alcohol units/day or ≥15 units/week based on the study questionnaire.

Comorbidities were ascertained from medical history, current medications, and/or newly established diagnoses during the index outpatient visit. Hypertension was defined as systolic blood pressure ≥140 mmHg and/or diastolic blood pressure ≥90 mmHg, and/or current use of antihypertensive medication.[13] Coronary artery disease (CAD) was defined as a prior history of acute coronary syndrome or the presence of chest pain accompanied by abnormalities on a routine 12-lead electrocardiogram.[14] Congestive heart failure (CHF) was defined by symptoms consistent with heart failure together with abnormal findings on echocardiography.[15] Overall cardiovascular disease (CVD) comprised CAD, CHF, and stroke. Diabetes mellitus was defined according to the American Diabetes Association criteria using any of the following: fasting plasma glucose ≥126 mg/dL (7.0 mmol/L), HbA1c ≥6.5% or random plasma glucose ≥200 mg/dL with classic hyperglycemic symptoms.[16] Chronic kidney disease was defined according to KDIGO

as abnormalities of kidney structure or function present for ≥3 months with health implications.[17] Chronic liver disease in this study referred to cirrhosis only (no other chronic liver conditions were recorded).

Data analysis

All analyses were conducted in R (R Foundation for Statistical Computing, Vienna, Austria).[18] Data were cleaned and managed using the dplyr and tidyr packages. Baseline characteristics were summarized using tableone and/or gtsummary: continuous variables are presented as mean ± standard deviation and compared between groups using the independent-samples t test; categorical variables are presented as n (%) and compared using the chi-square test, while exact methods were applied when appropriate (Fisher's exact test or the Fisher–Freeman–Halton exact test, implemented via stats and DescTools). The prevalence of osteosarcopenia across comorbid conditions was calculated with 95% confidence intervals (CIs) for proportions (Wilson score method, via binom), and visualized using column charts with error bars (95% CIs) created with ggplot2. Associations with osteosarcopenia were first examined using univariable logistic regression, followed by multivariable

logistic regression models. Variables with $p < 0.20$ in univariable analyses were entered into multivariable models,[19] statistical significance was defined as two-sided $p < 0.05$. Model results were reported as odds ratios (ORs) with 95% CIs, extracted and formatted using broom, and displayed as forest plots of adjusted ORs using ggplot2 (with ggforestplot or forestplot). To clarify the role of CVD, two multivariable models were fitted: one including overall CVD together with other selected covariates, and a second replacing overall CVD with its individual components while retaining the remaining covariates. Although overall CVD remained significant in the composite model, component-level results were additionally reported to identify specific CVD conditions associated with osteosarcopenia.

Results

Among the 389 participants, 80.2% were women. The overall prevalence of osteosarcopenia was 26.7% (95% CI 22.6%–31.3%), with similar rates in men (28.6%, 95% CI 19.7%–39.5%) and women (26.3%, 95% CI 21.7%–31.4%). Age, gait speed, and the prevalence of low physical performance did not differ significantly between sexes (all p >

0.05). In contrast, men had significantly higher handgrip strength, appendicular skeletal muscle mass and index, as well as greater total hip and femoral neck BMD than women (all $p < 0.001$).

Table 1. Age and musculoskeletal characteristics of the study participants

Characteristics	Men (n = 77)	Women (n = 312)	Total (n = 389)	p
Age (years), mean (SD)	71.71 (8.29)	69.93 (6.97)	70.29 (7.28)	0.085[a]
**Muscle strength**				
Max handgrip strength (kg), mean (SD)	27.03 (8.10)	19.81 (4.71)	21.28 (6.25)	<0.001[a]
Low muscle strength, n (%)	33 (42.9)	88 (28.2)	121 (31.1)	0.013[b]
**Physical performance**				
Gait speed (m/s), mean (SD)	0.9 (0.2)	0.8 (0.3)	0.84 (0.32)	0.159[a]
Low physical performance, n (%)	43 (55.8)	205 (65.7)	248 (63.8)	0.107[b]
**Muscle mass**				
ASM (kg), mean (SD)	17.76 (3.03)	13.00 (2.23)	13.93 (3.05)	<0.001[a]
ASMI (kg/m^2), mean (SD)	6.73 (0.95)	5.59 (0.84)	5.81 (0.98)	<0.001[a]

Low appendicular skeletal muscle mass, n (%)	51 (66.2)	131 (42.0)	182 (46.8)	<0.001[b]
**Bone mineral density**				
Total hip BMD (g/cm²), mean (SD)	0.93 (0.13)	0.76 (0.12)	0.79 (0.14)	<0.001[a]
Femoral neck BMD (g/cm²), mean (SD)	0.87 (0.15)	0.70 (0.12)	0.73 (0.14)	<0.001[a]
BMD classification, n (%)				<0.001[b]
Normal	37 (48.1)	47 (15.1)	84 (21.6)	
Low bone mass	8 (10.4)	126 (40.4)	134 (34.4)	
Osteoporosis	32 (41.6)	139 (44.6)	171 (44.0)	

[a]*Independent Sample T test,*
[b]*Chi-square test*

Compared with participants without osteosarcopenia, those with osteosarcopenia were older ($p = 0.004$) and had lower BMI ($p < 0.001$). The prevalence of hypertension, congestive heart failure, cardiovascular disease, and diabetes was significantly higher in the osteosarcopenia group (all $p \leq 0.040$), whereas sex distribution, living arrangement, smoking and alcohol use, fall and fracture history, family history of fracture, glucocorticoid use, coronary artery disease, stroke, dyslipidemia, chronic kidney disease, and chronic liver disease did not differ

significantly between groups (all $p > 0.05$). Bone mineral density classification differed substantially, with a predominance of osteoporosis in the osteosarcopenia group ($p < 0.001$), and sarcopenia was present in all individuals with osteosarcopenia by definition ($p < 0.001$).

Table 2. Baseline characteristics of participants according to osteosarcopenia status

Characteristics	OS (n = 104)	Non-OS (n = 285)	Total (n = 389)	p
Male, n (%)	22 (21.2)	55 (19.3)	77 (19.8)	0.684[a]
Age (years), mean (SD)	72.03 (6.88)	69.65 (7.32)	70.29 (7.28)	**0.004[b]**
Weight (kg)	48.70 (6.57)	58.70 (8.52)	56.03 (9.18)	**<0.001[b]**
Height (m)	1.53 (0.07)	1.54 (0.07)	1.54 (0.07)	0.485[b]
BMI (kg/m^2), mean (SD)	20.58 (2.56)	24.60 (3.16)	23.53 (3.50)	**<0.001[b]**
BMI Classification, n (%)				**<0.001[c]**
Underweight	22 (21.2)	4 (1.4)	26 (6.7)	
Normal weight	64 (61.5)	89 (31.2)	153 (39.3)	
Overweight	14 (13.5)	81 (28.4)	95 (24.4)	
Obesity	4 (3.8)	111 (38.9)	115 (29.6)	
Living arrangement, n (%)				0.242[a]
With family	90 (86.5)	252 (73.7)	342 (87.9)	

Alone	15 (14.4)	29 (10.2)	44 (11.3)	
Smoking history, n (%)	14 (13.5)	33 (11.6)	47 (12.1)	0.614[a]
Alcohol use, n (%)	11 (10.6)	27 (9.5)	38 (9.8)	0.746[a]
Fall history, n (%)	22 (21.2)	53 (18.6)	75 (19.3)	0.571[a]
Fracture history, n (%)	16 (15.4)	25 (8.8)	41 (10.5)	0.060[a]
Family history of fracture, n (%)	6 (5.8)	9 (3.2)	15 (3.9)	0.236[a]
Glucocorticoids use, n (%)	32 (30.8)	79 (27.7)	111 (28.5)	0.555[a]
Hypertension, n (%)	58 (55.8)	120 (42.1)	178 (45.8)	**0.017[a]**
CAD, n (%)	33 (31.7)	63 (22.1)	96 (24.7)	0.051[a]
CHF, n (%)	4 (4.8)	2 (0.7)	6 (1.5)	**0.040[d]**
Stroke, n (%)	3 (2.9)	2 (0.7)	5 (1.3)	0.121[d]
CVD, n (%)	39 (37.5)	66 (23.2)	105 (27.0)	**0.005[a]**
Diabetes, n (%)	45 (43.3)	72 (25.3)	117 (30.1)	**<0.001[a]**
Dyslipidemia, n (%)	36 (34.6)	87 (30.5)	123 (31.6)	0.443[a]
Chronic kidney disease, n (%)	8 (7.7)	15 (5.3)	23 (5.9)	0.369[a]
Chronic liver disease, n (%)	2 (1.9)	8 (2.8)	10 (2.6)	1.000[a]
BMD classification, n (%)				**<0.001[c]**
Normal	0 (0.0)	84 (29.5)	84 (21.6)	
Osteopenia	46 (44.2)	125 (43.9)	171 (44.0)	
Osteoporosis	58 (55.8)	76 (26.7)	134 (34.4)	
Sarcopenia, n (%)	104 (100)	28 (9.8)	132 (33.9)	**<0.001[c]**

[a]Chi-square test, [b]Independent Sample T test, [c]Fisher Freeman Halton Exact test, [d]Fisher's Exact test

Figure 1. Prevalence of Osteosarcopenia Across Comorbid Conditions

Fig 1 shows that the prevalence of osteosarcopenia was highest among participants with sarcopenia (78.8%, 95% CI 71.1–84.9), followed by those with congestive heart failure (66.7%, 30.0–90.3) and prior stroke (60.0%, 23.1–88.2). In contrast, the lowest prevalence was observed in chronic liver disease (20.0%, 5.7–51.0). Across most cardiometabolic conditions, including hypertension, coronary artery disease, cardiovascular disease, diabetes, and dislipidemia, osteosarcopenia prevalence was consistently around one-third.

In univariate analysis, older age ($p = 0.004$), higher BMI ($p < 0.001$), hypertension ($p = 0.017$), cardiovascular disease ($p = 0.005$), diabetes ($p < 0.001$), and chronic heart failure ($p = 0.047$) were significantly associated with higher odds of osteosarcopenia.

Table 3. Univariate analysis associated factors for osteosarcopenia

Fators	Unit of comparison	OR (95% CI)	p
Male	Yes	1.12 (0.63–1.93)	0.684[a]
Age	+10 year	1.55 (1.14–2.10)	**0.004[b]**
BMI	+1 kg/m^2	0.59 (0.52–0.67)	**<0.001[b]**
Smoking history	Yes	1.19 (0.59–2.28)	0.614[a]
Alcohol use	Yes	1.13 (0.52–2.31)	0.746[a]
Glucocorticoids use	Yes	1.16 (0.70–1.88)	0.555[a]
Hypertension	Yes	1.73 (1.10–2.74)	**0.017[a]**
CAD	Yes	1.64 (0.99–2.69)	0.051[a]
Chronic heart failure	Yes	5.66 (1.09–41.28)	**0.047[d]**
Stroke	Yes	4.20 (0.69–32.25)	0.119[d]
Cardiovascular disease	Yes	1.99 (1.22–3.22)	**0.005[a]**
Diabetes	Yes	2.26 (1.41–3.62)	**<0.001[a]**
Dyslipidemia	Yes	1.20 (0.74–1.93)	0.443[a]
Chronic kidney disease	Yes	1.50 (0.59–3.57)	0.369[a]
Chronic liver disease	Yes	0.68 (0.10–2.76)	0.626[a]

Fig 2. Multivariate analysis associated factors for osteosarcopenia

In multivariable analysis (**Fig 2**), older age (per +10 years; OR 1.77, 95% CI 1.20–2.65; p = 0.004), higher BMI (per +1 kg/m^2; OR 0.57, 95%

CI 0.49–0.64; $p < 0.001$), coronary artery disease (OR 2.12, 95% CI 1.11–4.09; $p = 0.024$), and diabetes (OR 2.60, 95% CI 1.41–4.86; $p = 0.002$) remained independently associated with osteosarcopenia. In contrast, hypertension, chronic heart failure, and stroke were not independently associated after adjustment (all $p > 0.05$).

Discussions

In this cross-sectional analysis of older Vietnamese outpatients, osteosarcopenia was identified in more than one-quarter of the cohort (26.7%). The distribution was similar between sexes. Advanced age, lower body mass index, coronary artery disease, and diabetes mellitus were independently associated with osteosarcopenia. Collectively, these results underscore the substantial clinical relevance of osteosarcopenia in this population and indicate a strong association with advancing age, reduced adiposity, and cardiometabolic comorbidities.

The prevalence of osteosarcopenia (26.7%) observed in our cohort lies within the wide range reported globally. A recent meta-analysis estimated a pooled prevalence of approximately 21% among older adults,

situating our findings slightly above the overall average.[3] This difference may be attributable to the hospital-based nature of our sample, which likely encompassed individuals with a higher burden of comorbid conditions. In contrast to many prior studies documenting a predominance of osteosarcopenia among women,[5] we identified comparable prevalence rates between sexes. Although osteoporosis is generally more common in women,[20] age-related decline in skeletal muscle mass and function among men may attenuate sex disparities in the combined phenotype of bone and muscle loss.[21] Furthermore, the lack of a significant difference in osteosarcopenia prevalence between men and women in our cohort may be attributable to the relatively small number of male participants, which could have limited statistical power, or to specific demographic and clinical features unique to our study population.

Consistent with prior research, increasing age was strongly associated with OS.[1] The co-occurrence of osteoporosis and sarcopenia in OS is believed to stem from convergent age-related mechanisms. With advancing age, immune and endocrine dysregulation (immunosenescence, sex hormone deficiencies, secondary

hyperparathyroidism, etc.) lead to simultaneous bone and muscle catabolism.[22, 23] There is also direct biochemical crosstalk between musculoskeletal tissues, whereby muscle-derived myokines contribute to the regulation of bone formation, and bone-derived osteokines modulate muscle metabolism.[24] In osteosarcopenia, these signaling pathways become impaired. Additionally, fatty infiltration of muscle and bone marrow increases with age and inactivity, causing local lipotoxic effects that further weaken both tissues.[25] This explains why poor nutrition and sedentarism are major risk factors for OS in the elderly.[8] The association of low BMI with OS in our study likely reflects the impact of chronic undernutrition and frailty – insufficient protein/calorie intake leads to loss of both bone density and muscle mass. Conversely, higher BMI can be protective to a point (indicating greater muscle and bone loading); however, the protective role of obesity is not uniform, and BMI alone may not confer protection, with fracture risk varying depending on whether excess weight reflects lean mass or fat mass.[3, 26] Moreover, our identification of diabetes and coronary artery disease as independent correlates of OS aligns with evidence linking chronic cardiometabolic

diseases to concurrent bone and muscle loss.[27] For instance, Moretti et al. (2022) reported that post-menopausal women with type 2 diabetes had about five times higher odds of osteosarcopenia than non-diabetics.[28] Patients with long-standing diabetes often exhibit both reduced bone strength and accelerated muscle wasting due to hyperglycemia-induced tissue damage and hormonal resistance.[28] Similarly, atherosclerotic cardiovascular disease contributes to a chronic pro-inflammatory milieu and reduced physical activity, both of which may accelerate musculoskeletal deterioration. These pathophysiological considerations support the concept that osteosarcopenia arises from interconnected biological mechanisms rather than the simple coexistence of two independent disorders. In our analysis, hypertension and heart failure showed significant associations with osteosarcopenia in univariate models; however, these relationships were no longer evident after multivariable adjustment, indicating that their effects may be confounded or mediated by age and other covariates. Taken together, our findings reinforce the multifactorial pathogenesis of osteosarcopenia described in

previous studies, while also suggesting potential population-specific patterns within our cohort.

Our findings further emphasize that osteosarcopenia constitutes a clinically significant geriatric syndrome that requires early recognition and integrated management strategies. Older individuals presenting with concomitant low bone mass and reduced muscle quantity or function represent a particularly vulnerable subgroup. Evidence from international cohorts indicates that patients with osteosarcopenia face markedly increased risks of falls, fragility fractures, and premature mortality compared with those affected by either osteoporosis or sarcopenia alone.[1] From a clinical perspective, the coexistence of bone fragility and poor muscle function greatly increases the risk of serious events such as hip fractures, which are linked to high mortality, long-term disability, and substantial healthcare costs. Therefore, clinicians should remain alert to the possibility of osteosarcopenia in older adults, particularly those with chronic diseases, frailty, or declining physical performance. Simple screening tools, such as handgrip strength or usual gait speed to evaluate muscle function, together with bone mineral density measurement by

dual-energy X-ray absorptiometry, can be readily applied in primary care and geriatric settings to support early detection of this high-risk condition.[1, 29, 30] Early recognition enables timely intervention – optimal management of OS requires addressing both its components. Guidelines emphasize treating the bone aspect with appropriate osteoporosis medications (calcium/vitamin D supplementation and antiresorptive or anabolic therapies) while simultaneously implementing interventions for muscle strengthening, such as protein supplementation and progressive resistance exercise training.[31-34] Multidisciplinary measures including fall prevention strategies and physical therapy are also essential. In the context of Vietnam, increasing awareness of osteosarcopenia among healthcare providers is crucial so that at-risk older adults (for example, those with diabetes or cardiovascular disease) receive integrated bone-muscle health assessments and interventions. Such an approach has the potential to reduce fractures, preserve independence, and improve overall quality of life in our aging population.

This study contributes additional data on osteosarcopenia in Vietnam and, to our knowledge, is among the first to examine its prevalence and

associated factors in a Vietnamese population. A key strength is the application of established diagnostic criteria, including AWGS 2019 for sarcopenia and WHO definitions for low bone mass, together with objective DXA measurements to enhance the accuracy of case classification. Nevertheless, several limitations should be acknowledged. First, the cross-sectional design precludes causal inference, and the observed associations cannot clarify temporal relationships. For instance, it remains uncertain whether specific comorbidities contribute to the development of osteosarcopenia or whether underlying osteosarcopenia predisposes individuals to these conditions. Second, the study population was recruited from a single-center outpatient clinic and may not fully represent the broader elderly population. Older adults seeking hospital-based care may differ systematically in health status from community-dwelling peers. In addition, the exclusion of individuals with advanced diseases, such as end-stage renal disease or recent stroke, as well as those receiving osteoporosis treatment, was intended to reduce confounding but may have led to an underestimation of prevalence by omitting more frail or previously treated patients. Third, the predominance of women in the

cohort (approximately 80%) and the relatively limited number of male participants may have reduced statistical power to detect sex-specific differences. Furthermore, we did not directly assess dietary intake, or inflammatory biomarkers, which could have provided further insight into the mechanisms underlying osteosarcopenia. Finally, given the hospital-based design, caution is warranted when extrapolating these findings to other clinical settings or ethnic populations. Despite these constraints, our study provides foundational data on osteosarcopenia in Vietnam and underscores priorities for future research.

Conclusion

Osteosarcopenia was a common condition in this cohort of older Vietnamese outpatients, affecting approximately one in four participants. Advanced age, low body mass index, coronary artery disease, and type 2 diabetes mellitus were identified as independent factors associated with osteosarcopenia. These findings highlight the need for routine screening for combined bone and muscle loss in high-risk older adults and the implementation of integrated management strategies to improve patient outcomes, especially in high-risk individuals.

Acknowledgments

We are grateful to express our sincere gratitude to the Rectorate Board of University of Medicine and Pharmacy, Hue University and Can Tho University of Medicine and Pharmacy for creating favorable conditions for this study to be carried out.

Author contributions

Conception and design of the study: Long H. Ngo, Huong T. T. Hoang, Van T. H. Nguyen; Data analysis and interpretation of data: Long H. Ngo, Hoa T. Nguyen, An V. Tran; Draft the manuscript: Long H. Ngo. Revise the manuscript: Long H. Ngo, An V. Tran, Huong T. T. Hoang, Van T. H. Nguyen. Final Approval: Long H. Ngo, Huong T. T. Hoang, Van T. H. Nguyen, Hoa T. Nguyen, An V. Tran.

References

1. Kirk B, Zanker J, Duque G. Osteosarcopenia: epidemiology, diagnosis, and treatment-facts and numbers. *J Cachexia Sarcopenia Muscle*. 11 (2020) 609-618. doi:10.1002/jcsm.12567.
2. Paintin J, Cooper C, Dennison E. Osteosarcopenia. *Br J Hosp Med (Lond)*. 79 (2018) 253-258. doi:10.12968/hmed.2018.79.5.253.
3. Huang T, Li C, Chen F, Xie D, Yang C, Chen Y, et al. Prevalence and risk factors of osteosarcopenia: a systematic review and meta-analysis. *BMC Geriatr*. 23 (2023) 369. doi:10.1186/s12877-023-04085-9.
4. Polito A, Barnaba L, Ciarapica D, Azzini E. Osteosarcopenia: A Narrative Review on Clinical Studies. *Int J Mol Sci*. 23 (2022) 10.3390/ijms23105591.

5. Inoue T, Maeda K, Nagano A, Shimizu A, Ueshima J, Murotani K, et al. Related Factors and Clinical Outcomes of Osteosarcopenia: A Narrative Review. *Nutrients*. 13 (2021) 10.3390/nu13020291.
6. Hoang DK, Doan MC, Mai LD, Ho-Le TP, Ho-Pham LT. Burden of osteoporosis in Vietnam: An analysis of population risk. *PLoS One*. 16 (2021) e0252592. doi:10.1371/journal.pone.0252592.
7. Hoang DK, Doan MC, Le NM, Nguyen HG, Ho-Pham LT, Nguyen TV. Prevalence of and risk factors for sarcopenia in community-dwelling people: The Vietnam Osteoporosis Study. *J Cachexia Sarcopenia Muscle*. 15 (2024) 380-386. doi:10.1002/jcsm.13383.
8. Kirk B, Miller S, Zanker J, Duque G. A clinical guide to the pathophysiology, diagnosis and treatment of osteosarcopenia. *Maturitas*. 140 (2020) 27-33. doi:10.1016/j.maturitas.2020.05.012.
9. Slart R, Punda M, Ali DS, Bazzocchi A, Bock O, Camacho P, et al. Updated practice guideline for dual-energy X-ray absorptiometry (DXA). *Eur J Nucl Med Mol Imaging*. 52 (2025) 539-563. doi:10.1007/s00259-024-06912-6.
10. Camacho PM, Petak SM, Binkley N, Diab DL, Eldeiry LS, Farooki A, et al. American Association of Clinical Endocrinologists/American College of Endocrinology clinical practice guidelines for the diagnosis and treatment of postmenopausal osteoporosis—2020 Update. *Endocr Pract*. 26 (2020) 1-46. doi:10.4158/gl-2020-0524suppl.
11. Chen L-K, Woo J, Assantachai P, Auyeung T-W, Chou M-Y, Iijima K, et al. Asian Working Group for Sarcopenia: 2019 Consensus Update on Sarcopenia Diagnosis and Treatment. *Journal of the American Medical Directors Association*. 21 (2020) 300-307.e2. doi:10.1016/j.jamda.2019.12.012.
12. Bajaj SS, Zhong A, Zhang AL, Stanford FC. Body Mass Index Thresholds for Asians: A Race Correction in Need of Correction? *Ann Intern Med*. 177 (2024) 1127-1129. doi:10.7326/m24-0161.
13. McEvoy JW, McCarthy CP, Bruno RM, Brouwers S, Canavan MD, Ceconi C, et al. 2024 ESC Guidelines for the management of elevated blood pressure and hypertension. *Eur Heart J*. 45 (2024) 3912-4018. doi:10.1093/eurheartj/ehae178.
14. Balakumar P, Kavitha M, Nanditha S. Cardiovascular drugs-induced oral toxicities: A murky area to be revisited and illuminated. *Pharmacol Res*. 102 (2015) 81-9. doi:10.1016/j.phrs.2015.09.007.
15. McDonagh TA, Metra M, Adamo M, Gardner RS, Baumbach A, Böhm M, et al. 2021 ESC Guidelines for the diagnosis and treatment of acute and chronic heart failure. *Eur Heart J*. 42 (2021) 3599-3726. doi:10.1093/eurheartj/ehab368.
16. American Diabetes Association Professional Practice Committee. 2. Diagnosis and Classification of Diabetes: Standards of Care in Diabetes-2025. *Diabetes Care*. 48 (2025) S27-s49. doi:10.2337/dc25-S002.
17. Kidney Disease: Improving Global Outcomes (KDIGO) CKD Work Group. KDIGO 2024 Clinical Practice Guideline for the Evaluation and Management of Chronic Kidney Disease. *Kidney Int*. 105 (2024) S117-s314. doi:10.1016/j.kint.2023.10.018.
18. R Core Team. A language and environment for statistical computing. Foundation for Statistical Computing, Vienna, Austria. https://www.R-project.org/
19. Malhotra RK. Errors in the use of Multivariable Logistic Regression Analysis: An Empirical Analysis. *Indian J Community Med*. 45 (2020) 560-562. doi:10.4103/ijcm.IJCM_16_20.

20. Cosman F, de Beur SJ, LeBoff MS, Lewiecki EM, Tanner B, Randall S, et al. Clinician's Guide to Prevention and Treatment of Osteoporosis. *Osteoporos Int*. 25 (2014) 2359-81. doi:10.1007/s00198-014-2794-2.
21. Cruz-Jentoft AJ, Bahat G, Bauer J, Boirie Y, Bruyère O, Cederholm T, et al. Sarcopenia: revised European consensus on definition and diagnosis. *Age Ageing*. 48 (2019) 601. doi:10.1093/ageing/afz046.
22. Kirk B, Al Saedi A, Duque G. Osteosarcopenia: A case of geroscience. *Aging Med (Milton)*. 2 (2019) 147-156. doi:10.1002/agm2.12080.
23. Hirschfeld HP, Kinsella R, Duque G. Osteosarcopenia: where bone, muscle, and fat collide. *Osteoporos Int*. 28 (2017) 2781-2790. doi:10.1007/s00198-017-4151-8.
24. Hamrick MW. A role for myokines in muscle-bone interactions. *Exerc Sport Sci Rev*. 39 (2011) 43-7. doi:10.1097/JES.0b013e318201f601.
25. Al Saedi A, Hassan EB, Duque G. The diagnostic role of fat in osteosarcopenia. *Journal of Laboratory and Precision Medicine*. 4 (2019).
26. Nguyen HT, Nguyen BT, Nguyen QT, Tran AV, Thai NHT, Phan QXT, et al. Muscle mass, not fat mass, predicts vertebral fracture risk: Vietnam osteoporosis study. *Osteoporos Int*. (2026) 10.1007/s00198-025-07831-4.
27. Park C-H, Lee Y-T, Yoon KJ. Association between osteosarcopenia and coronary artery calcification in asymptomatic individuals. *Scientific Reports*. 12 (2022) 2231. doi:10.1038/s41598-021-02640-1.
28. Moretti A, Palomba A, Gimigliano F, Paoletta M, Liguori S, Zanfardino F, et al. Osteosarcopenia and type 2 diabetes mellitus in post-menopausal women: a case-control study. *Orthop Rev (Pavia)*. 14 (2022) 38570. doi:10.52965/001c.38570.
29. Nguyen HT, Nguyen BT, Thai THN, Tran AV, Nguyen TT, Vo T, et al. Prevalence, incidence of and risk factors for vertebral fracture in the community: the Vietnam Osteoporosis Study. *Sci Rep*. 14 (2024) 32. doi:10.1038/s41598-023-50145-w.
30. Nguyen HT, Nguyen BT, Tran AV, Nguyen TT, Ngo LH, Vo T, et al. A predictive nomogram for selective screening of asymptomatic vertebral fractures: The Vietnam Osteoporosis Study. *Osteoporos Sarcopenia*. 11 (2025) 9-14. doi:10.1016/j.afos.2024.12.002.
31. Kirk B, Mooney K, Amirabdollahian F, Khaiyat O. Exercise and Dietary-Protein as a Countermeasure to Skeletal Muscle Weakness: Liverpool Hope University - Sarcopenia Aging Trial (LHU-SAT). *Front Physiol*. 10 (2019) 445. doi:10.3389/fphys.2019.00445.
32. Morton RW, Murphy KT, McKellar SR, Schoenfeld BJ, Henselmans M, Helms E, et al. A systematic review, meta-analysis and meta-regression of the effect of protein supplementation on resistance training-induced gains in muscle mass and strength in healthy adults. *Br J Sports Med*. 52 (2018) 376-384. doi:10.1136/bjsports-2017-097608.
33. Bauer JM, Verlaan S, Bautmans I, Brandt K, Donini LM, Maggio M, et al. Effects of a vitamin D and leucine-enriched whey protein nutritional supplement on measures of sarcopenia in older adults, the PROVIDE study: a randomized, double-blind, placebo-controlled trial. *J Am Med Dir Assoc*. 16 (2015) 740-7. doi:10.1016/j.jamda.2015.05.021.

34. Verlaan S, Maier AB, Bauer JM, Bautmans I, Brandt K, Donini LM, et al. Sufficient levels of 25-hydroxyvitamin D and protein intake required to increase muscle mass in sarcopenic older adults - The PROVIDE study. *Clin Nutr*. 37 (2018) 551-557. doi:10.1016/j.clnu.2017.01.005.

Figure

Click here to access/download;Figure;2. Figure 1.tif

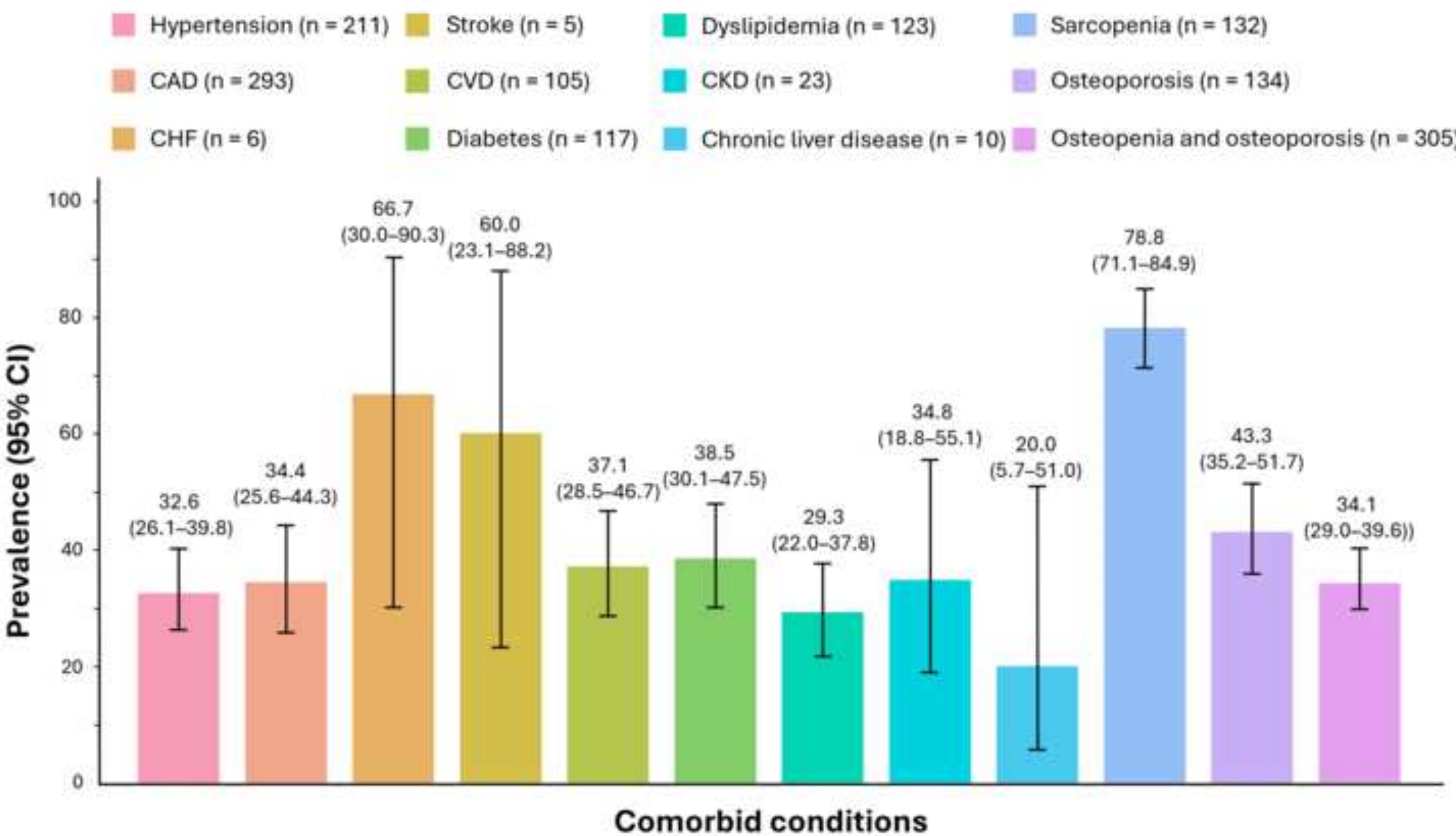

Figure
Click here to access/download;Figure;2b. Figure 2.tif

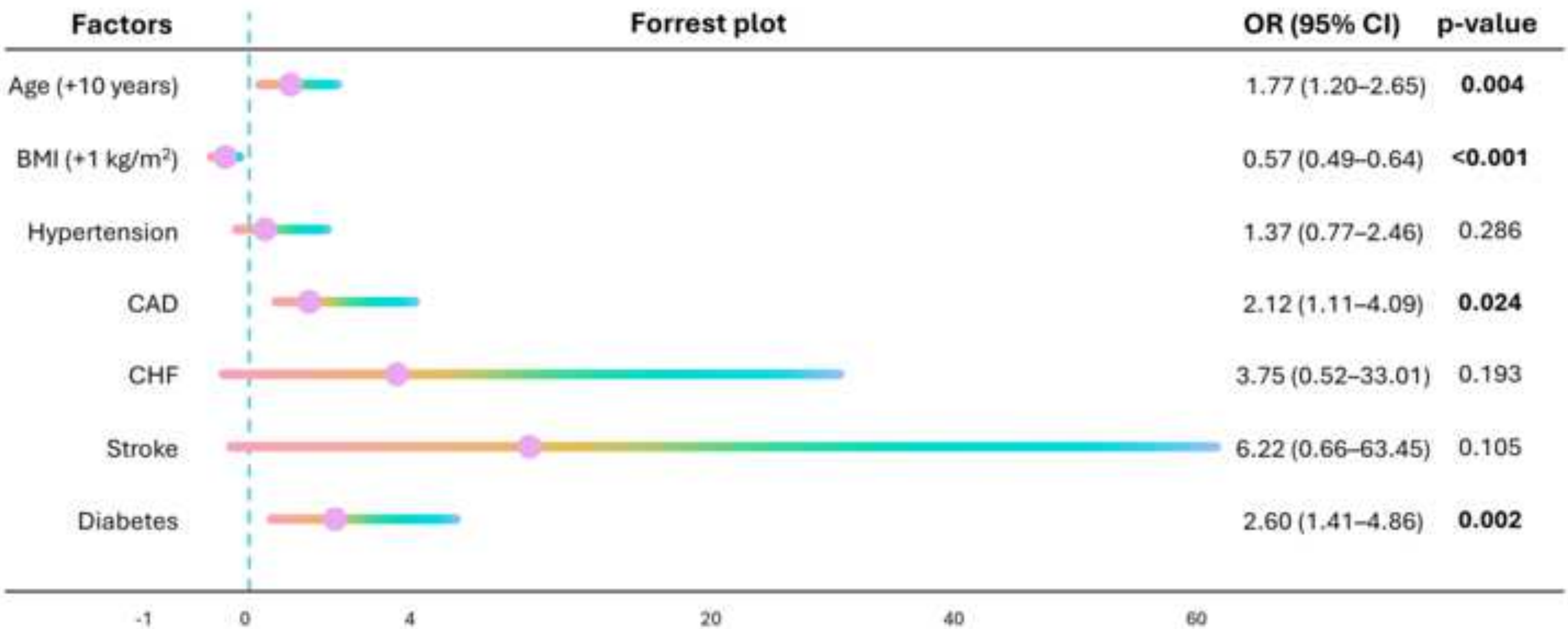

Click here to access/download
Supporting Information
6. Data osteosarcopenia in Elderly Vietnamese Adult.xlsx

Click here to access/download
Supporting Information
535-19.08.2024-Nguyen Hoang Thanh Van-Vietnamese.pdf
