# Supplementary material for: Prevalence of osteosarcopenia and associated factors among older Vietnamese outpatients: A cross-sectional study
Source: PLoS One. 2026 Oct 26;21(10):e0359850. doi: 10.1371/journal.pone.0359850 (PMC13704813; doi:10.1371/journal.pone.0359850)
Supplement: Supplementary file 2 [file pone.0359850.s001.docx]

**S1 Table. Age and musculoskeletal characteristics of the study participants**

| **Characteristics** | **Men**  **(n = 77)** | **Women**  **(n = 312)** | **Total**  **(n = 389)** | **p** |
| --- | --- | --- | --- | --- |
| Age (years), mean (SD) | 71.71 (8.29) | 69.93 (6.97) | 70.29 (7.28) | 0.085^a^ |
| **Muscle strength** | | | | |
| Max handgrip strength (kg), mean (SD) | 27.03 (8.10) | 19.81 (4.71) | 21.28 (6.25) | <0.001^a^ |
| Low muscle strength, n (%) | 33 (42.9) | 88 (28.2) | 121 (31.1) | 0.013^b^ |
| **Physical performance** | | | | |
| Gait speed (m/s), mean (SD) | 0.9 (0.2) | 0.8 (0.3) | 0.84 (0.32) | 0.159^a^ |
| Low physical performance, n (%) | 43 (55.8) | 205 (65.7) | 248 (63.8) | 0.107^b^ |
| **Muscle mass** | | | | |
| ASM (kg), mean (SD) | 17.76 (3.03) | 13.00 (2.23) | 13.93 (3.05) | <0.001^a^ |
| ASMI (kg/m^2^), mean (SD) | 6.73 (0.95) | 5.59 (0.84) | 5.81 (0.98) | <0.001^a^ |
| Low appendicular skeletal muscle mass, n (%) | 51 (66.2) | 131 (42.0) | 182 (46.8) | <0.001^b^ |
| **Bone mineral density** | | | | |
| Total hip BMD (g/cm²), mean (SD) | 0.93 (0.13) | 0.76 (0.12) | 0.79 (0.14) | <0.001^a^ |
| Femoral neck BMD (g/cm²), mean (SD) | 0.87 (0.15) | 0.70 (0.12) | 0.73 (0.14) | <0.001^a^ |
| BMD classification, n (%) |  |  |  | <0.001^b^ |
| T-score ≥ −1.0 | 37 (48.1) | 47 (15.1) | 84 (21.6) |  |
| −2.5 < T-score < −1.0 | 8 (10.4) | 126 (40.4) | 134 (34.4) |  |
| T-score ≤ −2.5 | 32 (41.6) | 139 (44.6) | 171 (44.0) |  |
| *^a^Independent Sample T test, ^b^Chi-square test* | | | | |
