# Supplementary material for: Prevalence of osteosarcopenia and associated factors among older Vietnamese outpatients: A cross-sectional study
Source: PLoS One. 2026 Oct 26;21(10):e0359850. doi: 10.1371/journal.pone.0359850 (PMC13704813; doi:10.1371/journal.pone.0359850)
Supplement: Supplementary file 3 [file pone.0359850.s002.docx]

**S2 Table. Sensitivity analysis using Firth penalized multivariable logistic regression including comorbidities with sparse cell counts**

| **Factors** | **Unit of comparison** | **Adjusted OR (95% CI)** | **p** |
| --- | --- | --- | --- |
| Age | Per 1-year increase | 1.06 (1.02–1.10) | **0.005** |
| BMI | Per 1 kg/m² increase | 0.58 (0.51–0.66) | **<0.001** |
| Fracture history | Yes | 1.33 (0.54–3.18) | 0.532 |
| Hypertension | Yes | 1.34 (0.75–2.37) | 0.322 |
| Coronary artery disease | Yes | 2.04 (1.08–3.90) | **0.029** |
| Congestive heart failure | Yes | 3.49 (0.54–24.82) | 0.186 |
| Stroke | Yes | 5.86 (0.69–48.21) | 0.103 |
| Chronic liver disease | Yes | 0.51 (0.05–3.67) | 0.538 |
| Diabetes mellitus | Yes | 2.54 (1.39–4.72) | **0.002** |
