# Supplementary material for: Prevalence of osteosarcopenia and associated factors among older Vietnamese outpatients: A cross-sectional study
Source: PLoS One. 2026 Oct 26;21(10):e0359850. doi: 10.1371/journal.pone.0359850 (PMC13704813; doi:10.1371/journal.pone.0359850)
Supplement: Supplementary file 5 [file pone.0359850.s005.docx]

Reviewer comments:

1. Uniformity in definition of Osteosarcopenia is needed. Some place it is mentioned as Osteoporosis + Sarcopenia and some place it is mentio0ned as Osteopenia & Osteoprosis + Sarcopenia
2. Selection bias : As population is visiting to hospital outpatients are considered, not general population
3. Gender Bias : Male to female ratio is skewed
4. Conclusion could be elderly population visiting hospital has higher prevalence of Osteosarcopenia.
