# Supplementary material for: Prevalence of osteosarcopenia and associated factors among older Vietnamese outpatients: A cross-sectional study
Source: PLoS One. 2026 Oct 26;21(10):e0359850. doi: 10.1371/journal.pone.0359850 (PMC13704813; doi:10.1371/journal.pone.0359850)
Supplement: Supplementary file 6 [file pone.0359850.s008.docx]

**Manuscript ID: PONE-D-26-13624**

**Manuscript title: Osteosarcopenia in Elderly Vietnamese Adult: Prevalence and Risk factors**

Dear Academic Editor and Reviewers,

We would like to sincerely thank the Academic Editor and the reviewers for their careful evaluation of our manuscript and for providing constructive comments and valuable suggestions. We have carefully revised the manuscript according to the comments raised during the review process. The changes have been incorporated into the revised manuscript, and a marked-up version with tracked changes has also been prepared. Below, we provide a point-by-point response to each comment. Reviewer comments are reproduced in bold, followed by our responses.

We hope that the revised manuscript has adequately addressed all concerns and meets the requirements for further consideration by PLOS ONE.

**Reviewer #1: Reviewer comments:**

**1. Uniformity in definition of Osteosarcopenia is needed. Some place it is mentioned as Osteoporosis + Sarcopenia and some place it is mentio0ned as Osteopenia & Osteoprosis + Sarcopenia**

**RESPONSE:** Thank you for this important comment. We agree that the definition of osteosarcopenia should be consistent throughout the manuscript. We have carefully revised the Abstract, Introduction, Methods, Discussion, and tables to standardize the terminology. In the revised manuscript, osteosarcopenia is uniformly defined as the coexistence of sarcopenia and low bone mass, including either osteopenia or osteoporosis. We also revised the BMD classification terminology in the tables to clearly distinguish osteopenia from osteoporosis according to T-score criteria.

**2. Selection bias: As population is visiting to hospital outpatients are considered, not general population**

**RESPONSE:** Thank you for this important comment. We agree that the hospital-based outpatient recruitment may introduce selection bias and limit the generalizability of our findings to the general elderly population. We have revised the Limitations section to clarify that older adults seeking hospital-based care may differ from community-dwelling older adults in terms of health status and comorbidity burden. We also added that the observed prevalence should be interpreted as reflecting older hospital outpatients rather than the general elderly population.

**3. Gender Bias: Male to female ratio is skewed**

**RESPONSE:** Thank you for this valuable comment. We agree that the unbalanced sex distribution, with a predominance of female participants, may introduce gender-related bias and may affect the interpretation of the overall prevalence estimate and sex-specific comparisons. We have revised the Limitations section to acknowledge that the predominance of women and the relatively limited number of male participants may have reduced the statistical power to detect sex-specific differences. We also clarified that this unbalanced sex distribution should be considered when interpreting the study findings.
**4. Conclusion could be elderly population visiting hospital has higher prevalence of Osteosarcopenia.**

**RESPONSE:** Thank you for this helpful suggestion. We agree that the conclusion should more clearly reflect the hospital-based outpatient population of our study and avoid overgeneralization to the general elderly population. We have revised the Conclusion section to state that osteosarcopenia was common among older Vietnamese adults visiting a hospital outpatient clinic, rather than referring broadly to the general elderly population.

**Reviewer #2: 1- page 20- table low bone mass means osteopenia ;you can mention t-Score values**

**RESPONSE:** Thank you for this helpful comment. We agree that the term “low bone mass” in the BMD classification table may be ambiguous, particularly because it can be interpreted broadly as including both osteopenia and osteoporosis. To avoid confusion, we have revised the table terminology by replacing “low bone mass” with “osteopenia” where appropriate. We have also added the corresponding T-score thresholds in the Methods section and table footnote to clarify the BMD classification. Osteopenia was defined as a T-score between −1.0 and −2.5, whereas osteoporosis was defined as a T-score ≤ −2.5, using the lowest T-score among the lumbar spine, total hip, and femoral neck.

**2-page 24 multivariate analysis -Increase bmi is associated with osteosarcpenia but forestplot showing protective one, explain it.**

**RESPONSE:** Thank you for this helpful comment. We agree that the previous wording may have been unclear and could have led to misinterpretation. In both the univariate and multivariable analyses, BMI showed an odds ratio below 1, indicating an inverse association with osteosarcopenia. Therefore, higher BMI was associated with lower odds of osteosarcopenia, whereas lower BMI was associated with higher odds of osteosarcopenia. We have revised the Results section to clearly describe this inverse association and to ensure consistency with the forest plot. We also refined the relevant text in the Discussion and Conclusion sections to avoid any ambiguity in the interpretation of BMI.
