# Supplementary material for: Prevalence of osteosarcopenia and associated factors among older Vietnamese outpatients: A cross-sectional study
Source: PLoS One. 2026 Oct 26;21(10):e0359850. doi: 10.1371/journal.pone.0359850 (PMC13704813; doi:10.1371/journal.pone.0359850)
Supplement: Supplementary file 7 [file pone.0359850.s009.docx]

**Manuscript ID: PONE-D-26-13624**

**Manuscript title: Prevalence of Osteosarcopenia and Associated Factors Among Older Vietnamese Outpatients: A Cross-Sectional Study**

Dear Academic Editor and Reviewers,

We would like to sincerely thank the Academic Editor and the reviewers for their careful evaluation of our manuscript and for providing constructive comments and valuable suggestions. We have carefully revised the manuscript according to the comments raised during the review process. The changes have been incorporated into the revised manuscript, and a marked-up version with tracked changes has also been prepared. Below, we provide a point-by-point response to each comment. Reviewer comments are reproduced in bold, followed by our responses.

We hope that the revised manuscript has adequately addressed all concerns and meets the requirements for further consideration by PLOS ONE.

**# ADDITIONAL EDITOR COMMENTS:**

**1. Please review all PLOS One submission guidelines. I note that references need to be formatted according to the guidelines, and age and line numbers should be added. In addition, although many of the relevant details are included in the paper, I encourage the authors to include a completed STROBE checklist for cross-sectional studies. Please include details on the sample size calculation, and provide more details on the participant sampling/recruitment (i.e., what was the size of the target population, how many people were invited to participate and how were they recruited? How many were excluded based on the listed criteria? A flow diagram may help to illustrate how the authors arrived at the final sample size, and this could be moved to the results).**

**RESPONSE:** Thank you for this comprehensive comment. We have carefully reviewed the PLOS ONE submission guidelines and revised the manuscript accordingly. The references have been reformatted in accordance with the journal’s Vancouver style, and page numbers and continuous line numbers have been added throughout the revised manuscript. We have also completed the STROBE checklist for cross-sectional studies and submitted it as Supporting Information.

Regarding the sample size, it was determined during the development of the original study protocol using the single-proportion formula: $\text{n}\text{ }\text{=}\text{ }\frac{\text{Z}_{\text{1-}\frac{\text{α}}{\text{2}}}^{\text{2}}\text{ }\text{×}\text{ }\text{p}\text{ }\text{×}\text{ }\text{(1-p)}}{\text{d}^{\text{2}}}$. A prevalence of 42% was selected based on a previous Vietnamese study involving 243 older inpatients, as this was the most directly relevant local estimate available when the protocol was developed. Using a 95% confidence level and an absolute precision of 5% (d = 0.05), the minimum required sample size was 375 participants. Ultimately, 389 participants were included in the final analysis. The local study used to obtain the planning estimate was published in a Vietnamese-language journal, involved a relatively small single-center inpatient sample, and did not have a DOI or international indexing (<https://sti.gov.vn/publication/ti-le-dong-mac-thieu-co-loang-xuong-va-mot-so-yeu-to-lien-quan-o-nguoi-benh-cao-tuoi-dieu-tri-noi-tru-tai-khoa-lao-hoc-benh-vien-nhan-dan-gia-dinh-MzYzNzA3>). Because the prevalence estimate used for the sample size calculation was derived from a locally published Vietnamese study that does not have a DOI and is not internationally indexed, we were uncertain whether including this source in the manuscript reference list would be consistent with the journal’s referencing expectations and were concerned about its accessibility and verifiability for international readers. Therefore, in the current revision, we have provided the Editor with the complete basis and calculation of the sample size for transparency. In the manuscript, we have added a participant flow diagram to clearly present the recruitment process and show how the final analytical sample of 389 participants was obtained. However, if the Editor considers it necessary, we would be pleased to add the sample size calculation and the corresponding local reference to the Methods in accordance with the journal’s requirements.

To clarify the participant sampling and recruitment process, potential participants were identified and approached during their routine outpatient visits. During the study period, 913 patients aged ≥60 years were screened for participation. Of these, 278 declined to participate and 213 were excluded based on the eligibility criteria: inability to ambulate or perform the required physical assessments (n = 34), residual deficits from ischemic or hemorrhagic stroke (n = 78), a history of limb amputation (n = 12), and current or prior osteoporosis treatment (n = 89). The remaining 422 eligible participants underwent handgrip-strength and gait-speed assessments. Subsequently, 33 declined DXA scanning, leaving 389 participants who completed all required assessments and were included in the final analysis. These details are summarized in the participant flow diagram added as Fig 1 in the revised manuscript.

**2. Regarding the analysis, the results presented in Table 1 are not relevant unless there was a study objective related to whether sex differences were apparent in any of the key outcomes. Regarding the univariate logistic regression analysis, I question the validity of the results for those variables with very small cell sizes such as CHF, stroke and chronic liver disease. In addition, some of the variables (fall & fracture history, family history of fracture) were not mentioned in the methods text. I am also unclear why the authors chose to use 10-year age groupings and without providing the age range of the cohort, and the number of participants in the 10-year age groupings it is difficult to interpret this finding.**

**RESPONSE:** Thank you for raising these important points.

First, we agree that the investigation of sex differences was not a prespecified objective of this study. However, the muscle- and bone-related measurements presented in the former Table 1 constitute the diagnostic components used to characterize sarcopenia and low bone mass, which together define osteosarcopenia. We therefore considered their descriptive presentation relevant to the interpretation of the study outcome. Nevertheless, we have reduced the emphasis placed on these descriptive results in the main manuscript and moved the former Table 1 to S1 Table.

Second, we rechecked the cell counts for congestive heart failure (n = 6), stroke (n = 5), and chronic liver disease (n = 10). We acknowledge that conventional maximum-likelihood estimates for these sparse conditions may be unstable and should be interpreted cautiously. We retained these variables in the univariable analyses for exploratory completeness and reported their odds ratios with 95% confidence intervals to demonstrate the imprecision of the estimates. However, they were not included in the primary conventional multivariable logistic regression model because their small cell counts could produce unstable adjusted estimates. Instead, these sparse comorbidities were evaluated in a sensitivity analysis using Firth penalized multivariable logistic regression. The results, presented in S2 Table, were consistent with those of the primary model, although the estimates for the sparse comorbidities remained imprecise, with wide confidence intervals.

Third, we have added information regarding the ascertainment and binary coding of fall history, fracture history, and family history of fracture to the Methods section. These variables have also been included in the revised univariable analysis.

Finally, age has been re-expressed as a continuous variable per 1-year increase rather than per 10-year increase throughout the Abstract, Methods, Results, tables, and figure. The observed age range of the cohort (60–96 years) has also been added.

**3. The figures have captions in the text but cannot stand alone from the text because they provide insufficient information (i.e., for Figure 2, the caption should clarify that the figure presents the adjusted odds ratios (95% CI) for risk factors associated with osteosarcopenia, include the sample size and the reference categories, and indicate what the shading refers to. For the tables, as I mentioned above, Table 1 isn’t relevant unless there is an objective related to examining sex differences. For table 2, age can be reported to 1 decimal place, and the same is likely true for height, weight and BMI assuming methods used to measure height and weight were only accurate to one decimal place. Also, in Table 2, the sum of 252 + 29 for living arrangement in the Non-OS group doesn’t equal 285, and the categories Normal, Osteopenia, Osteporosis, should include the T-scores as was done for Table 1.**

**RESPONSE:** Thank you for these detailed comments.

We have revised the captions of both figures so that they can be understood independently of the main text. The caption for Figure 2 now clarifies that the columns represent the prevalence of osteosarcopenia across comorbid conditions and that the error bars represent 95% confidence intervals. The caption for Figure 3 now specifies that the figure presents adjusted odds ratios with 95% confidence intervals from the primary multivariable logistic regression model. It also reports the sample size (n = 389, including 104 participants with osteosarcopenia and 285 without osteosarcopenia), identifies the reference categories for categorical variables, and specifies the units of comparison for age and BMI. The caption further explains that the points represent adjusted odds ratios, the horizontal lines represent 95% confidence intervals, and the vertical dashed line at an odds ratio of 1.0 indicates no association. P-values <0.05 are shown in bold.

As described in our response above, we have reduced the emphasis placed on the descriptive sex-stratified results in the main manuscript and moved the former Table 1 to S1 Table.

In the former Table 2, now renumbered as Table 1 in the main manuscript, age, weight, and BMI have been reported to one decimal place.

We also rechecked the source data for living arrangement and identified a transcription error. The correct numbers of participants living with family are 89 in the osteosarcopenia group and 256 in the non-osteosarcopenia group, giving a total of 345 participants. The corresponding numbers living alone are 15 and 29, respectively, giving a total of 44 participants. The table has been corrected accordingly, and the p-value remains unchanged at 0.242.

Finally, the T-score thresholds have been added to the bone mineral density categories as follows: normal, T-score ≥ −1.0; osteopenia, −2.5 < T-score < −1.0; and osteoporosis, T-score ≤ −2.5. The spelling of “osteoporosis” has also been corrected.

**4. Overall, considering the methodological limitations noted above it is difficult to determine whether the conclusions are fully supported by the data.**

**RESPONSE:** Thank you for this important observation. We have carefully reconsidered and revised the interpretation and conclusions in light of the methodological limitations raised. Given the cross-sectional design, we have removed causal terminology such as “risk factors” and now consistently describe the findings as associations with prevalent osteosarcopenia. We have also removed the composite CVD variable from all descriptive and regression analyses, excluded comorbidities with sparse cell counts from the primary conventional multivariable logistic regression model, and evaluated these sparse variables using Firth penalized logistic regression as a sensitivity analysis. In addition, we have added a participant flow diagram detailing the recruitment process, clarified the statistical methods and regression diagnostics, and strengthened the Limitations regarding the single-center outpatient sample, sex imbalance, potential selection bias, and unmeasured nutritional, vitamin D, calcium-intake, physical-activity, and inflammatory factors. Finally, the Conclusion has been revised to emphasize the prevalence and observed associations within this outpatient population without implying causality or generalizability to the broader older population. We believe that these revisions provide a more cautious interpretation that is appropriately supported by the study design and data.

**Other comments**

**1. Intro: Please provide a reference for the definition of osteosarcopenia (first sentence).**

**RESPONSE:** Thank you for this comment. A relevant reference has been added to support the definition of osteosarcopenia in the first sentence of the Introduction.

**2. Intro, Para 3: Provide a reference to support the statement that “Most studies to date have been conducted in Western populations…” and also for the statement that ends with “…investigated in epidemiological research.”**

**RESPONSE:** Thank you for this comment. We have added relevant references to support the geographical distribution of previous osteosarcopenia studies and the epidemiological investigation of osteoporosis in Vietnam.

**3. Intro, last paragraph: The final sentence would be better placed in the discussion / conclusion.**

**RESPONSE:** Thank you for this suggestion. We have removed the final sentence from the Introduction and moved it to the final paragraph of the Discussion, where it more appropriately highlights the broader contribution of the study.

**4. Methods, Study Setting: Why is “Viet Nam” used here but “Vietnam” is used in the introduction?**

**RESPONSE:** Thank you for noting this inconsistency. We have standardized the spelling to “Vietnam” throughout the manuscript.

**5. Variables: Please provide the units for ASM and aBMD at first mention, and also clarify that BMD by DXA is an areal measurement. Please also clarify which hip was scanned, how many technicians acquired & analyzed the scans, and the %CV for the ASM measurement.**

**RESPONSE:** Thank you for this comment. We have revised the Methods to provide the units of ASM (kg) and DXA-derived areal bone mineral density (BMD; g/cm²) at their first mention. We have also clarified that hip BMD was measured at the femoral neck and total hip on the non-dominant side. The non-dominant leg was defined as the leg not selected as the supporting leg during a one-leg standing test. The dominant hip was assessed when a femoral head prosthesis or metallic implant was present on the non-dominant side. All scans were acquired by one trained DXA technologist and subsequently reviewed and analyzed by one investigator. The coefficients of variation were 2.0% for ASM, 1.8% for lumbar spine BMD, and 1.5% for proximal femur BMD. These details have been added to the revised Methods.

**6. Sarcopenia: Please provide more details on AWGS criteria for sarcopenia, as it isn’t clear how the various measures taken in this study were used to determine sarcopenia, and it would help if the reader didn’t have to refer to the AWGS publication for those details.**

**RESPONSE:** Thank you for this comment. We have clarified how the individual AWGS 2019 measures were combined to determine sarcopenia. Specifically, sarcopenia was diagnosed when low ASMI was accompanied by low muscle strength, low physical performance, or both, while severe sarcopenia required the presence of all three components. Participants meeting the criteria for either sarcopenia or severe sarcopenia were considered to have sarcopenia when defining osteosarcopenia.

**7. Consider changing “Baseline characteristics” to “Participant characteristics” or “Sociodemographic characteristics”, as baseline implies that this was a prospective study. Also, the first sentence of this section only mentions sex, age, BMI and living arrangement but alcohol and smoking are also mentioned. In addition, please clarify if height and weight were measured by study staff or if BMI was calculated based on self-reported height and weight. Also, how close was the “index outpatient visit” to the time of the study measurements?**

**RESPONSE:** Thank you for this comment. We have changed the subsection title from “Baseline characteristics” to “Participant characteristics” to better reflect the cross-sectional study design. We have also clarified the data-collection procedure. Upon arrival at the outpatient clinic, participants’ height and weight were measured by clinic nurses as part of the routine assessment and were not self-reported; BMI was subsequently calculated as weight in kilograms divided by height in meters squared. Patients were then invited to participate in the study, and those who provided informed consent underwent all remaining study assessments during the same outpatient visit. To avoid ambiguity, we replaced the term “index outpatient visit” with “study enrollment visit” and clarified that this visit occurred on the same day as the study assessments.

**8. Data analysis: Please clarify why the prevalence of osteosarcopenia across comorbid conditions was assessed, as only general prevalence was mentioned in the study objective. In addition, the statement “Associations with osteosarcopenia…” is rather vague – I assume you mean associations between potential risk factors and prevalence of osteosarcopenia? Regarding the models with CVD – I don’t see results for two models presented. I’m also unclear why overall CVD would be included in a model with the individual components, as the composite variable wouldn’t carry any independent information. Please also clarify how regression diagnostics were checked.**

**RESPONSE:** Thank you for these important comments. We retained the analysis of osteosarcopenia prevalence across comorbid conditions because these condition-specific estimates provide clinically relevant information and may highlight patient groups with a higher burden of osteosarcopenia who could benefit from more proactive screening and assessment. To align the study objective with the analyses performed, we revised the objective to explicitly include the estimation of both overall prevalence and prevalence across these selected conditions. We agree that the phrase “associations with osteosarcopenia” was insufficiently specific. Our intention was to examine the associations between potential explanatory factors and prevalent osteosarcopenia. Given the cross-sectional design, we reviewed the manuscript throughout and replaced causal terminology, including “risk factors,” with non-causal wording such as “associated factors” and “factors associated with prevalent osteosarcopenia.”

Our initial rationale for evaluating overall cardiovascular disease (CVD) was to combine participants with individual cardiovascular conditions and thereby improve statistical precision. However, after reconsidering this approach, we found that coronary artery disease (CAD) accounted for 96 of the 105 participants originally classified as having overall CVD. Therefore, the composite CVD variable provided little additional information beyond CAD and substantially overlapped with its individual component. We consequently removed overall CVD from all descriptive and regression analyses, deleted the previous description of two CVD models, and retained CAD as the cardiovascular variable in the primary multivariable model. Congestive heart failure and stroke, with only six and five cases, respectively, were retained in the exploratory univariable analyses but were not included in the primary conventional multivariable model because of their sparse cell counts. These conditions were subsequently evaluated in the Firth penalized multivariable logistic regression sensitivity analysis.

We also clarified the regression diagnostic procedures. All variance inflation factors were below 1.05, indicating no meaningful multicollinearity. The Hosmer–Lemeshow test showed no evidence of poor model fit (p = 0.109), and no observation had a Cook’s distance greater than 1, suggesting that no individual observation exerted undue influence on the model estimates. The functional forms of the continuous predictors were examined using the Box–Tidwell approach and a spline-based sensitivity analysis. BMI showed no evidence of departure from linearity, whereas a possible nonlinear association between age and prevalent osteosarcopenia was observed. Nevertheless, the spline-based sensitivity analysis yielded similar findings for the principal associated factors and did not alter the main conclusions. These revisions and diagnostic procedures have been clarified in the Data Analysis section.

**9. Results: The term “rates” implies a time dimension – please remove this term from the results and discussion. P-values that are presented in the tables do not need to be repeated in the text.**

**RESPONSE:** Thank you for this comment. We have removed the term “rates” and used “prevalence” consistently throughout the Results and Discussion, as appropriate for the cross-sectional study design. We have also removed p-values from the narrative Results where they were already presented in the corresponding tables or figure, while retaining the principal findings and effect estimates with 95% confidence intervals where informative.

**10. Discussion: Since osteosarcopenia is spelled out in full elsewhere in the manuscript, I would suggest not using the abbreviation in the discussion.**

**RESPONSE:** Thank you for this suggestion. We have replaced all instances of the abbreviation “OS” with the full term “osteosarcopenia” throughout the Discussion.

**11. Discussion, limitations: Consider adding physical activity to the list of outcomes not assessed in this study, but which might provide further insight into mechanisms underpinning osteosarcopenia.**

**RESPONSE:** Thank you for this suggestion. We have added physical activity to the limitations, together with other relevant factors that were not assessed. We have also noted that evaluating physical activity may provide further insight into the mechanisms underlying osteosarcopenia.

**# REVIEWER 2**

**You have not mentioned the subjects' nutritional status, calcium uptake, and vitamin D status, which are relevant to the evaluation of osteosarcopenia. Have you used the physical activity (PA) questionnaire to assess PA?**

**RESPONSE:** Thank you for this important comment. Detailed nutritional status beyond BMI, dietary calcium intake, and vitamin D status were not assessed in this study. In addition, a validated physical activity questionnaire was not administered. We have acknowledged the absence of these potentially relevant factors in the Limitations and noted that they should be considered in future studies.
