# Supplementary material for: Prevalence of osteosarcopenia and associated factors among older Vietnamese outpatients: A cross-sectional study
Source: PLoS One. 2026 Oct 26;21(10):e0359850. doi: 10.1371/journal.pone.0359850 (PMC13704813; doi:10.1371/journal.pone.0359850)
Supplement: Peer review history

# Peer review: Decision Letter 0

**DOI:** 10.1371/journal.pone.0359850.r001

**Authors:** Rimesh Pal

2 May 2026

Dear Dr. Nguyen,

Thank you for submitting your manuscript to PLOS ONE. After careful consideration, we feel that it has merit but does not fully meet PLOS ONE’s publication criteria as it currently stands. Therefore, we invite you to submit a revised version of the manuscript that addresses the points raised during the review process.

Please submit your revised manuscript by Jun 16 2026 11:59PM. If you will need more time than this to complete your revisions, please reply to this message or contact the journal office at plosone@plos.org. When you're ready to submit your revision, log on to https://www.editorialmanager.com/pone/ and select the 'Submissions Needing Revision' folder to locate your manuscript file.

- A letter that responds to each point raised by the academic editor and reviewer(s). You should upload this letter as a separate file labeled 'Response to Reviewers'.
- A marked-up copy of your manuscript that highlights changes made to the original version. You should upload this as a separate file labeled 'Revised Manuscript with Track Changes'.
- An unmarked version of your revised paper without tracked changes. You should upload this as a separate file labeled 'Manuscript'.

If you would like to make changes to your financial disclosure, please include your updated statement in your cover letter. Guidelines for resubmitting your figure files are available below the reviewer comments at the end of this letter.

If applicable, we recommend that you deposit your laboratory protocols in protocols.io to enhance the reproducibility of your results. Protocols.io assigns your protocol its own identifier (DOI) so that it can be cited independently in the future. For instructions see: https://journals.plos.org/plosone/s/submission-guidelines#loc-laboratory-protocols. Additionally, PLOS ONE offers an option for publishing peer-reviewed Lab Protocol articles, which describe protocols hosted on protocols.io. Read more information on sharing protocols at https://plos.org/protocols?utm_medium=editorial-email&utm_source=authorletters&utm_campaign=protocols.

As the corresponding author, your ORCID iD is verified in the submission system and will appear in the published article. PLOS supports the use of ORCID, and we encourage all coauthors to register for an ORCID iD and use it as well. Please encourage your coauthors to verify their ORCID iD within the submission system before final acceptance, as unverified ORCID iDs will not appear in the published article. *Only*  the individual author can complete the verification step; PLOS staff *cannot*  verify ORCID iDs on behalf of authors.

We look forward to receiving your revised manuscript.

Kind regards,

Rimesh Pal

Academic Editor

PLOS One

Journal Requirements:

When submitting your revision, we need you to address these additional requirements.

1.Please ensure that your manuscript meets PLOS ONE's style requirements, including those for file naming. The PLOS ONE style templates can be found at

https://journals.plos.org/plosone/s/file?id=wjVg/PLOSOne_formatting_sample_main_body.pdf and

https://journals.plos.org/plosone/s/file?id=ba62/PLOSOne_formatting_sample_title_authors_affiliations.pdf

2. Thank you for providing your underlying data as Supporting Information.

We note that the data set contains text or data that is not in English. Please note that PLOS is an English-language publisher, so we require data sets to be provided in English as well. Please upload an English-language version of your data set.

This will also allow us to determine if your data follows PLOS standards per our Data Availability policy here: https://journals.plos.org/plosone/s/data-availability.

3. We note that there is identifying data in the Supporting Information file “6. Data osteosarcopenia in Elderly Vietnamese Adult.xlsx” Due to the inclusion of these potentially identifying data, we have removed this file from your file inventory. Prior to sharing human research participant data, authors should consult with an ethics committee to ensure data are shared in accordance with participant consent and all applicable local laws.

Data sharing should never compromise participant privacy. It is therefore not appropriate to publicly share personally identifiable data on human research participants. The following are examples of data that should not be shared:

-Name, initials, physical address

-Ages more specific than whole numbers

-Internet protocol (IP) address

-Specific dates (birth dates, death dates, examination dates, etc.)

-Contact information such as phone number or email address

-Location data

-ID numbers that seem specific (long numbers, include initials, titled “Hospital ID”) rather than random (small numbers in numerical order)

Data that are not directly identifying may also be inappropriate to share, as in combination they can become identifying. For example, data collected from a small group of participants, vulnerable populations, or private groups should not be shared if they involve indirect identifiers (such as sex, ethnicity, location, etc.) that may risk the identification of study participants.

Additional guidance on preparing raw data for publication can be found in our Data Policy (https://journals.plos.org/plosone/s/data-availability#loc-human-research-participant-data-and-other-sensitive-data) and in the following article: http://www.bmj.com/content/340/bmj.c181.long.

Please remove or anonymize all personal information (<specific identifying information in file to be removed>), ensure that the data shared are in accordance with participant consent, and re-upload a fully anonymized data set. Please note that spreadsheet columns with personal information must be removed and not hidden as all hidden columns will appear in the published file.

4. Please include your full ethics statement in the ‘Methods’ section of your manuscript file. In your statement, please include the full name of the IRB or ethics committee who approved or waived your study, as well as whether or not you obtained informed written or verbal consent. If consent was waived for your study, please include this information in your statement as well.

5. Please include captions for your Supporting Information files at the end of your manuscript, and update any in-text citations to match accordingly. Please see our Supporting Information guidelines for more information: http://journals.plos.org/plosone/s/supporting-information.

6. If the reviewer comments include a recommendation to cite specific previously published works, please review and evaluate these publications to determine whether they are relevant and should be cited. There is no requirement to cite these works unless the editor has indicated otherwise.

7. Please review your reference list to ensure that it is complete and correct. If you have cited papers that have been retracted, please include the rationale for doing so in the manuscript text, or remove these references and replace them with relevant current references. Any changes to the reference list should be mentioned in the rebuttal letter that accompanies your revised manuscript. If you need to cite a retracted article, indicate the article’s retracted status in the References list and also include a citation and full reference for the retraction notice.

Additional Editor Comments:

Please address the queries put forward by the reviewers.

[Note: HTML markup is below. Please do not edit.]

Reviewer's Responses to Questions

**Comments to the Author**

1. Is the manuscript technically sound, and do the data support the conclusions?

Reviewer #1: Yes

Reviewer #2: Yes

**********

2. Has the statistical analysis been performed appropriately and rigorously?

Reviewer #1: Yes

Reviewer #2: Yes

**********

3. Have the authors made all data underlying the findings in their manuscript fully available?

The PLOS Data policy

Reviewer #1: Yes

Reviewer #2: Yes

**********

4. Is the manuscript presented in an intelligible fashion and written in standard English?

Reviewer #1: Yes

Reviewer #2: Yes

**********

Reviewer #1: Reviewer comments:

1. Uniformity in definition of Osteosarcopenia is needed. Some place it is mentioned as Osteoporosis + Sarcopenia and some place it is mentio0ned as Osteopenia & Osteoprosis + Sarcopenia

2. Selection bias : As population is visiting to hospital outpatients are considered, not general population

3. Gender Bias : Male to female ratio is skewed

4. Conclusion could be elderly population visiting hospital has higher prevalence of Osteosarcopenia.

Reviewer #2: 1- page 20- table low bone mass means osteopenia ;you can mention t-Score values

2-page 24 multivariate analysis -Increase bmi is associated with osteosarcpenia but forestplot showing protective one ,explain it.

**********

what does this mean?). If published, this will include your full peer review and any attached files.

If you choose “no”, your identity will remain anonymous but your review may still be made public.

**Do you want your identity to be public for this peer review?**  For information about this choice, including consent withdrawal, please see our Privacy Policy

Reviewer #1: **Yes:**  DR. VIVEK ARYA

Reviewer #2: **Yes:**  Kishore Kumar Behera

**********

[NOTE: If reviewer comments were submitted as an attachment file, they will be attached to this email and accessible via the submission site. Please log into your account, locate the manuscript record, and check for the action link "View Attachments". If this link does not appear, there are no attachment files.]

To ensure your figures meet our technical requirements, please review our figure guidelines: https://journals.plos.org/plosone/s/figures

You may also use PLOS’s free figure tool, NAAS, to help you prepare publication quality figures: https://journals.plos.org/plosone/s/figures#loc-tools-for-figure-preparation.

NAAS will assess whether your figures meet our technical requirements by comparing each figure against our figure specifications.

Attachment Submitted filename: Reviewer comments-PLOS.docx

Attachment Submitted filename: plose one notes.pdf

# Author comment: Author response to Decision Letter 1

**DOI:** 10.1371/journal.pone.0359850.r002

24 Jun 2026

Manuscript ID: PONE-D-26-13624

Manuscript title: Osteosarcopenia in Elderly Vietnamese Adult: Prevalence and Risk factors

Dear Academic Editor and Reviewers,

We would like to sincerely thank the Academic Editor and the reviewers for their careful evaluation of our manuscript and for providing constructive comments and valuable suggestions. We have carefully revised the manuscript according to the comments raised during the review process. The changes have been incorporated into the revised manuscript, and a marked-up version with tracked changes has also been prepared. Below, we provide a point-by-point response to each comment. Reviewer comments are reproduced in bold, followed by our responses.

We hope that the revised manuscript has adequately addressed all concerns and meets the requirements for further consideration by PLOS ONE.

Reviewer #1: Reviewer comments:

1. Uniformity in definition of Osteosarcopenia is needed. Some place it is mentioned as Osteoporosis + Sarcopenia and some place it is mentio0ned as Osteopenia & Osteoprosis + Sarcopenia

RESPONSE: Thank you for this important comment. We agree that the definition of osteosarcopenia should be consistent throughout the manuscript. We have carefully revised the Abstract, Introduction, Methods, Discussion, and tables to standardize the terminology. In the revised manuscript, osteosarcopenia is uniformly defined as the coexistence of sarcopenia and low bone mass, including either osteopenia or osteoporosis. We also revised the BMD classification terminology in the tables to clearly distinguish osteopenia from osteoporosis according to T-score criteria.

2. Selection bias: As population is visiting to hospital outpatients are considered, not general population

RESPONSE: Thank you for this important comment. We agree that the hospital-based outpatient recruitment may introduce selection bias and limit the generalizability of our findings to the general elderly population. We have revised the Limitations section to clarify that older adults seeking hospital-based care may differ from community-dwelling older adults in terms of health status and comorbidity burden. We also added that the observed prevalence should be interpreted as reflecting older hospital outpatients rather than the general elderly population.

3. Gender Bias: Male to female ratio is skewed

RESPONSE: Thank you for this valuable comment. We agree that the unbalanced sex distribution, with a predominance of female participants, may introduce gender-related bias and may affect the interpretation of the overall prevalence estimate and sex-specific comparisons. We have revised the Limitations section to acknowledge that the predominance of women and the relatively limited number of male participants may have reduced the statistical power to detect sex-specific differences. We also clarified that this unbalanced sex distribution should be considered when interpreting the study findings.

4. Conclusion could be elderly population visiting hospital has higher prevalence of Osteosarcopenia.

RESPONSE: Thank you for this helpful suggestion. We agree that the conclusion should more clearly reflect the hospital-based outpatient population of our study and avoid overgeneralization to the general elderly population. We have revised the Conclusion section to state that osteosarcopenia was common among older Vietnamese adults visiting a hospital outpatient clinic, rather than referring broadly to the general elderly population.

Reviewer #2: 1- page 20- table low bone mass means osteopenia ;you can mention t-Score values

RESPONSE: Thank you for this helpful comment. We agree that the term “low bone mass” in the BMD classification table may be ambiguous, particularly because it can be interpreted broadly as including both osteopenia and osteoporosis. To avoid confusion, we have revised the table terminology by replacing “low bone mass” with “osteopenia” where appropriate. We have also added the corresponding T-score thresholds in the Methods section and table footnote to clarify the BMD classification. Osteopenia was defined as a T-score between −1.0 and −2.5, whereas osteoporosis was defined as a T-score ≤ −2.5, using the lowest T-score among the lumbar spine, total hip, and femoral neck.

2-page 24 multivariate analysis -Increase bmi is associated with osteosarcpenia but forestplot showing protective one, explain it.

RESPONSE: Thank you for this helpful comment. We agree that the previous wording may have been unclear and could have led to misinterpretation. In both the univariate and multivariable analyses, BMI showed an odds ratio below 1, indicating an inverse association with osteosarcopenia. Therefore, higher BMI was associated with lower odds of osteosarcopenia, whereas lower BMI was associated with higher odds of osteosarcopenia. We have revised the Results section to clearly describe this inverse association and to ensure consistency with the forest plot. We also refined the relevant text in the Discussion and Conclusion sections to avoid any ambiguity in the interpretation of BMI.

Attachment Submitted filename: Response to Reviewers.docx

# Peer review: Decision Letter 1

**DOI:** 10.1371/journal.pone.0359850.r003

**Authors:** Heather Macdonald

25 Aug 2026

Dear Dr. Nguyen,

Thank you for submitting your manuscript to PLOS One. After careful consideration, we feel that it has merit but does not fully meet PLOS One’s publication criteria as it currently stands. Therefore, we invite you to submit a revised version of the manuscript that addresses the points raised during the review process.

Please submit your revised manuscript by Oct 09 2026 11:59PM. If you will need more time than this to complete your revisions, please reply to this message or contact the journal office at plosone@plos.org. When you're ready to submit your revision, log on to https://www.editorialmanager.com/pone/ and select the 'Submissions Needing Revision' folder to locate your manuscript file.

- A letter that responds to each point raised by the academic editor and reviewer(s). You should upload this letter as a separate file labeled 'Response to Reviewers'.
- A marked-up copy of your manuscript that highlights changes made to the original version. You should upload this as a separate file labeled 'Revised Manuscript with Track Changes'.
- An unmarked version of your revised paper without tracked changes. You should upload this as a separate file labeled 'Manuscript'.

If you would like to make changes to your financial disclosure, please include your updated statement in your cover letter. Guidelines for resubmitting your figure files are available below the reviewer comments at the end of this letter.

If applicable, we recommend that you deposit your laboratory protocols in protocols.io to enhance the reproducibility of your results. Protocols.io assigns your protocol its own identifier (DOI) so that it can be cited independently in the future. For instructions see: https://journals.plos.org/plosone/s/submission-guidelines#loc-laboratory-protocols. Additionally, PLOS One offers an option for publishing peer-reviewed Lab Protocol articles, which describe protocols hosted on protocols.io. Read more information on sharing protocols at https://plos.org/protocols?utm_medium=editorial-email&utm_source=authorletters&utm_campaign=protocols.

As the corresponding author, your ORCID iD is verified in the submission system and will appear in the published article. PLOS supports the use of ORCID, and we encourage all coauthors to register for an ORCID iD and use it as well. Please encourage your coauthors to verify their ORCID iD within the submission system before final acceptance, as unverified ORCID iDs will not appear in the published article. *Only*  the individual author can complete the verification step; PLOS staff *cannot*  verify ORCID iDs on behalf of authors.

We look forward to receiving your revised manuscript.

Kind regards,

Heather Macdonald, Ph.D

Academic Editor

PLOS One

Journal Requirements:

If the reviewer comments include a recommendation to cite specific previously published works, please review and evaluate these publications to determine whether they are relevant and should be cited. There is no requirement to cite these works unless the editor has indicated otherwise.

**Additional Editor Comments:**

Please note that I am a new AE on this manuscript as the previous AE was not available. I have a number of concerns that were not raised by the 2 reviewers.

1. Please review all PLOS One submission guidelines. I note that references need to be formatted according to the guidelines, and age and line numbers should be added. In addition, although many of the relevant details are included in the paper, I encourage the authors to include a completed STROBE checklist for cross-sectional studies. Please include details on the sample size calculation, and provide more details on the participant sampling / recruitment (i.e., what was the size of the target population, how many people were invited to participate and how were they recruited? How many were excluded based on the listed criteria? A flow diagram may help to illustrate how the authors arrived at the final sample size, and this could be moved to the results)

2. Regarding the analysis, the results presented in Table 1 are not relevant unless there was a study objective related to whether sex differences were apparent in any of the key outcomes. Regarding the univariate logistic regression analysis, I question the validity of the results for those variables with very small cell sizes such as CHF, stroke and chronic liver disease. In addition, some of the variables (fall & fracture history, family history of fracture) were not mentioned in the methods text. I am also unclear why the authors chose to use 10-year age groupings and without providing the age range of the cohort, and the number of participants in the 10-year age groupings it is difficult to interpret this finding.

3. The figures have captions in the text but cannot stand alone from the text because they provide insufficient information (i.e., for Figure 2, the caption should clarify that the figure presents the adjusted odds ratios (95% CI) for risk factors associated with osteosarcopenia, include the sample size and the reference categories, and indicate what the shading refers to. For the tables, as I mentioned above, Table 1 isn’t relevant unless there is an objective related to examining sex differences. For table 2, age can be reported to 1 decimal place, and the same is likely true for height, weight and BMI assuming methods used to measure height and weight were only accurate to one decimal place. Also, in Table 2, the sum of 252 + 29 for living arrangement in the Non-OS group doesn’t equal 285, and the categories Normal, Osteopenia, Osteporosis, should include the T-scores as was done for Table 1.

4. Overall, considering the methodological limitations noted above it is difficult to determine whether the conclusions are fully supported by the data.

Other comments

1. Intro: Please provide a reference for the definition of osteosarcopenia (first sentence).

2. Intro, Para 3: Provide a reference to support the statement that “Most studies to date have been conducted in Western populations…” and also for the statement that ends with “…investigated in epidemiological research.”

3. Intro, last paragraph: The final sentence would be better placed in the discussion / conclusion.

4. Methods, Study Setting: Why is “Viet Nam” used here but “Vietnam” is used in the introduction?

5. Variables: Please provide the units for ASM and aBMD at first mention, and also clarify that BMD by DXA is an areal measurement. Please also clarify which hip was scanned, how many technicians acquired & analyzed the scans, and the %CV for the ASM measurement.

6. Sarcopenia: Please provide more details on AWGS criteria for sarcopenia, as it isn’t clear how the various measures taken in this study were used to determine sarcopenia, and it would help if the reader didn’t have to refer to the AWGS publication for those details.

7. Consider changing “Baseline characteristics” to “Participant characteristics” or “Sociodemographic characteristics”, as baseline implies that this was a prospective study. Also, the first sentence of this section only mentions sex, age, BMI and living arrangement but alcohol and smoking are also mentioned. In addition, please clarify if height and weight were measured by study staff or if BMI was calculated based on self-reported height and weight. Also, how close was the “index outpatient visit” to the time of the study measurements?

8. Data analysis: Please clarify why the prevalence of osteosarcopenia across comorbid conditions was assessed, as only general prevalence was mentioned in the study objective. In addition, the statement “Associations with osteosarcopenia…” is rather vague – I assume you mean associations between potential risk factors and prevalence of osteosarcopenia? Regarding the models with CVD – I don’t see results for two models presented. I’m also unclear why overall CVD would be included in a model with the individual components, as the composite variable wouldn’t carry any independent information. Please also clarify how regression diagnostics were checked.

9. Results: The term “rates” implies a time dimension – please remove this term from the results and discussion. P-values that are presented in the tables do not need to be repeated in the text.

10. Discussion: Since osteosarcopenia is spelled out in full elsewhere in the manuscript, I would suggest not using the abbreviation in the discussion.

11. Discussion, limitations: Consider adding physical activity to the list of outcomes not assessed in this study, but which might provide further insight into mechanisms underpinning osteosarcopenia.

[Note: HTML markup is below. Please do not edit.]

Reviewers' comments:

Reviewer's Responses to Questions

**Comments to the Author**

Reviewer #1: All comments have been addressed

Reviewer #2: All comments have been addressed

**********

2. Is the manuscript technically sound, and do the data support the conclusions?

Reviewer #1: Yes

Reviewer #2: Yes

**********

3. Has the statistical analysis been performed appropriately and rigorously?

Reviewer #1: Yes

Reviewer #2: (No Response)

**********

4. Have the authors made all data underlying the findings in their manuscript fully available?

The PLOS Data policy

Reviewer #1: Yes

Reviewer #2: Yes

**********

5. Is the manuscript presented in an intelligible fashion and written in standard English?

Reviewer #1: Yes

Reviewer #2: Yes

**********

Reviewer #1: After suggested changes, it is acceptable. It has been edited and corrected as per suggestions. Language used is appropriate.

Reviewer #2: You have not mentioned the subjects' nutritional status, calcium uptake, and vitamin D status, which are relevant to the evaluation of osteosarcopenia. Have you used the physical activity(PA) questionnaire to assess PA?

**********

what does this mean?). If published, this will include your full peer review and any attached files.

If you choose “no”, your identity will remain anonymous but your review may still be made public.

**Do you want your identity to be public for this peer review?**  For information about this choice, including consent withdrawal, please see our Privacy Policy

Reviewer #1: **Yes:**  ARYA VIVEK VED PRAKASH

Reviewer #2: No

**********

[NOTE: If reviewer comments were submitted as an attachment file, they will be attached to this email and accessible via the submission site. Please log into your account, locate the manuscript record, and check for the action link "View Attachments". If this link does not appear, there are no attachment files.]

To ensure your figures meet our technical requirements, please review our figure guidelines: https://journals.plos.org/plosone/s/figures

You may also use PLOS’s free figure tool, NAAS, to help you prepare publication quality figures: https://journals.plos.org/plosone/s/figures#loc-tools-for-figure-preparation.

NAAS will assess whether your figures meet our technical requirements by comparing each figure against our figure specifications.

# Author comment: Author response to Decision Letter 2

**DOI:** 10.1371/journal.pone.0359850.r004

16 Sep 2026

Manuscript ID: PONE-D-26-13624

Manuscript title: Prevalence of Osteosarcopenia and Associated Factors Among Older Vietnamese Outpatients: A Cross-Sectional Study

Dear Academic Editor and Reviewers,

We would like to sincerely thank the Academic Editor and the reviewers for their careful evaluation of our manuscript and for providing constructive comments and valuable suggestions. We have carefully revised the manuscript according to the comments raised during the review process. The changes have been incorporated into the revised manuscript, and a marked-up version with tracked changes has also been prepared. Below, we provide a point-by-point response to each comment. Reviewer comments are reproduced in bold, followed by our responses.

We hope that the revised manuscript has adequately addressed all concerns and meets the requirements for further consideration by PLOS ONE.

# ADDITIONAL EDITOR COMMENTS:

1. Please review all PLOS One submission guidelines. I note that references need to be formatted according to the guidelines, and age and line numbers should be added. In addition, although many of the relevant details are included in the paper, I encourage the authors to include a completed STROBE checklist for cross-sectional studies. Please include details on the sample size calculation, and provide more details on the participant sampling/recruitment (i.e., what was the size of the target population, how many people were invited to participate and how were they recruited? How many were excluded based on the listed criteria? A flow diagram may help to illustrate how the authors arrived at the final sample size, and this could be moved to the results).

RESPONSE: Thank you for this comprehensive comment. We have carefully reviewed the PLOS ONE submission guidelines and revised the manuscript accordingly. The references have been reformatted in accordance with the journal’s Vancouver style, and page numbers and continuous line numbers have been added throughout the revised manuscript. We have also completed the STROBE checklist for cross-sectional studies and submitted it as Supporting Information.

Regarding the sample size, it was determined during the development of the original study protocol using the single-proportion formula: "n = " ("Z" _("1-" "α" /"2" )^"2" " × p × (1-p)" )/"d" ^"2" . A prevalence of 42% was selected based on a previous Vietnamese study involving 243 older inpatients, as this was the most directly relevant local estimate available when the protocol was developed. Using a 95% confidence level and an absolute precision of 5% (d = 0.05), the minimum required sample size was 375 participants. Ultimately, 389 participants were included in the final analysis. The local study used to obtain the planning estimate was published in a Vietnamese-language journal, involved a relatively small single-center inpatient sample, and did not have a DOI or international indexing (https://sti.gov.vn/publication/ti-le-dong-mac-thieu-co-loang-xuong-va-mot-so-yeu-to-lien-quan-o-nguoi-benh-cao-tuoi-dieu-tri-noi-tru-tai-khoa-lao-hoc-benh-vien-nhan-dan-gia-dinh-MzYzNzA3). Because the prevalence estimate used for the sample size calculation was derived from a locally published Vietnamese study that does not have a DOI and is not internationally indexed, we were uncertain whether including this source in the manuscript reference list would be consistent with the journal’s referencing expectations and were concerned about its accessibility and verifiability for international readers. Therefore, in the current revision, we have provided the Editor with the complete basis and calculation of the sample size for transparency. In the manuscript, we have added a participant flow diagram to clearly present the recruitment process and show how the final analytical sample of 389 participants was obtained. However, if the Editor considers it necessary, we would be pleased to add the sample size calculation and the corresponding local reference to the Methods in accordance with the journal’s requirements.

To clarify the participant sampling and recruitment process, potential participants were identified and approached during their routine outpatient visits. During the study period, 913 patients aged ≥60 years were screened for participation. Of these, 278 declined to participate and 213 were excluded based on the eligibility criteria: inability to ambulate or perform the required physical assessments (n = 34), residual deficits from ischemic or hemorrhagic stroke (n = 78), a history of limb amputation (n = 12), and current or prior osteoporosis treatment (n = 89). The remaining 422 eligible participants underwent handgrip-strength and gait-speed assessments. Subsequently, 33 declined DXA scanning, leaving 389 participants who completed all required assessments and were included in the final analysis. These details are summarized in the participant flow diagram added as Fig 1 in the revised manuscript.

2. Regarding the analysis, the results presented in Table 1 are not relevant unless there was a study objective related to whether sex differences were apparent in any of the key outcomes. Regarding the univariate logistic regression analysis, I question the validity of the results for those variables with very small cell sizes such as CHF, stroke and chronic liver disease. In addition, some of the variables (fall & fracture history, family history of fracture) were not mentioned in the methods text. I am also unclear why the authors chose to use 10-year age groupings and without providing the age range of the cohort, and the number of participants in the 10-year age groupings it is difficult to interpret this finding.

RESPONSE: Thank you for raising these important points.

First, we agree that the investigation of sex differences was not a prespecified objective of this study. However, the muscle- and bone-related measurements presented in the former Table 1 constitute the diagnostic components used to characterize sarcopenia and low bone mass, which together define osteosarcopenia. We therefore considered their descriptive presentation relevant to the interpretation of the study outcome. Nevertheless, we have reduced the emphasis placed on these descriptive results in the main manuscript and moved the former Table 1 to S1 Table.

Second, we rechecked the cell counts for congestive heart failure (n = 6), stroke (n = 5), and chronic liver disease (n = 10). We acknowledge that conventional maximum-likelihood estimates for these sparse conditions may be unstable and should be interpreted cautiously. We retained these variables in the univariable analyses for exploratory completeness and reported their odds ratios with 95% confidence intervals to demonstrate the imprecision of the estimates. However, they were not included in the primary conventional multivariable logistic regression model because their small cell counts could produce unstable adjusted estimates. Instead, these sparse comorbidities were evaluated in a sensitivity analysis using Firth penalized multivariable logistic regression. The results, presented in S2 Table, were consistent with those of the primary model, although the estimates for the sparse comorbidities remained imprecise, with wide confidence intervals.

Third, we have added information regarding the ascertainment and binary coding of fall history, fracture history, and family history of fracture to the Methods section. These variables have also been included in the revised univariable analysis.

Finally, age has been re-expressed as a continuous variable per 1-year increase rather than per 10-year increase throughout the Abstract, Methods, Results, tables, and figure. The observed age range of the cohort (60–96 years) has also been added.

3. The figures have captions in the text but cannot stand alone from the text because they provide insufficient information (i.e., for Figure 2, the caption should clarify that the figure presents the adjusted odds ratios (95% CI) for risk factors associated with osteosarcopenia, include the sample size and the reference categories, and indicate what the shading refers to. For the tables, as I mentioned above, Table 1 isn’t relevant unless there is an objective related to examining sex differences. For table 2, age can be reported to 1 decimal place, and the same is likely true for height, weight and BMI assuming methods used to measure height and weight were only accurate to one decimal place. Also, in Table 2, the sum of 252 + 29 for living arrangement in the Non-OS group doesn’t equal 285, and the categories Normal, Osteopenia, Osteporosis, should include the T-scores as was done for Table 1.

RESPONSE: Thank you for these detailed comments.

We have revised the captions of both figures so that they can be understood independently of the main text. The caption for Figure 2 now clarifies that the columns represent the prevalence of osteosarcopenia across comorbid conditions and that the error bars represent 95% confidence intervals. The caption for Figure 3 now specifies that the figure presents adjusted odds ratios with 95% confidence intervals from the primary multivariable logistic regression model. It also reports the sample size (n = 389, including 104 participants with osteosarcopenia and 285 without osteosarcopenia), identifies the reference categories for categorical variables, and specifies the units of comparison for age and BMI. The caption further explains that the points represent adjusted odds ratios, the horizontal lines represent 95% confidence intervals, and the vertical dashed line at an odds ratio of 1.0 indicates no association. P-values <0.05 are shown in bold.

As described in our response above, we have reduced the emphasis placed on the descriptive sex-stratified results in the main manuscript and moved the former Table 1 to S1 Table.

In the former Table 2, now renumbered as Table 1 in the main manuscript, age, weight, and BMI have been reported to one decimal place.

We also rechecked the source data for living arrangement and identified a transcription error. The correct numbers of participants living with family are 89 in the osteosarcopenia group and 256 in the non-osteosarcopenia group, giving a total of 345 participants. The corresponding numbers living alone are 15 and 29, respectively, giving a total of 44 participants. The table has been corrected accordingly, and the p-value remains unchanged at 0.242.

Finally, the T-score thresholds have been added to the bone mineral density categories as follows: normal, T-score ≥ −1.0; osteopenia, −2.5 < T-score < −1.0; and osteoporosis, T-score ≤ −2.5. The spelling of “osteoporosis” has also been corrected.

4. Overall, considering the methodological limitations noted above it is difficult to determine whether the conclusions are fully supported by the data.

RESPONSE: Thank you for this important observation. We have carefully reconsidered and revised the interpretation and conclusions in light of the methodological limitations raised. Given the cross-sectional design, we have removed causal terminology such as “risk factors” and now consistently describe the findings as associations with prevalent osteosarcopenia. We have also removed the composite CVD variable from all descriptive and regression analyses, excluded comorbidities with sparse cell counts from the primary conventional multivariable logistic regression model, and evaluated these sparse variables using Firth penalized logistic regression as a sensitivity analysis. In addition, we have added a participant flow diagram detailing the recruitment process, clarified the statistical methods and regression diagnostics, and strengthened the Limitations regarding the single-center outpatient sample, sex imbalance, potential selection bias, and unmeasured nutritional, vitamin D, calcium-intake, physical-activity, and inflammatory factors. Finally, the Conclusion has been revised to emphasize the prevalence and observed associations within this outpatient population without implying causality or generalizability to the broader older population. We believe that these revisions provide a more cautious interpretation that is appropriately supported by the study design and data.

Other comments

1. Intro: Please provide a reference for the definition of osteosarcopenia (first sentence).

RESPONSE: Thank you for this comment. A relevant reference has been added to support the definition of osteosarcopenia in the first sentence of the Introduction.

2. Intro, Para 3: Provide a reference to support the statement that “Most studies to date have been conducted in Western populations…” and also for the statement that ends with “…investigated in epidemiological research.”

RESPONSE: Thank you for this comment. We have added relevant references to support the geographical distribution of previous osteosarcopenia studies and the epidemiological investigation of osteoporosis in Vietnam.

3. Intro, last paragraph: The final sentence would be better placed in the discussion / conclusion.

RESPONSE: Thank you for this suggestion. We have removed the final sentence from the Introduction and moved it to the final paragraph of the Discussion, where it more appropriately highlights the broader contribution of the study.

4. Methods, Study Setting: Why is “Viet Nam” used here but “Vietnam” is used in the introduction?

RESPONSE: Thank you for noting this inconsistency. We have standardized the spelling to “Vietnam” throughout the manuscript.

5. Variables: Please provide the units for ASM and aBMD at first mention, and also clarify that BMD by DXA is an areal measurement. Please also clarify which hip was scanned, how many technicians acquired & analyzed the scans, and the %CV for the ASM measurement.

RESPONSE: Thank you for this comment. We have revised the Methods to provide the units of ASM (kg) and DXA-derived areal bone mineral density (BMD; g/cm²) at their first mention. We have also clarified that hip BMD was measured at the femoral neck and total hip on the non-dominant side. The non-dominant leg was defined as the leg not selected as the supporting leg during a one-leg standing test. The dominant hip was assessed when a femoral head prosthesis or metallic implant was present on the non-dominant side. All scans were acquired by one trained DXA technologist and subsequently reviewed and analyzed by one investigator. The coefficients of variation were 2.0% for ASM, 1.8% for lumbar spine BMD, and 1.5% for proximal femur BMD. These details have been added to the revised Methods.

6. Sarcopenia: Please provide more details on AWGS criteria for sarcopenia, as it isn’t clear how the various measures taken in this study were used to determine sarcopenia, and it would help if the reader didn’t have to refer to the AWGS publication for those details.

RESPONSE: Thank you for this comment. We have clarified how the individual AWGS 2019 measures were combined to determine sarcopenia. Specifically, sarcopenia was diagnosed when low ASMI was accompanied by low muscle strength, low physical performance, or both, while severe sarcopenia required the presence of all three components. Participants meeting the criteria for either sarcopenia or severe sarcopenia were considered to have sarcopenia when defining osteosarcopenia.

7. Consider changing “Baseline characteristics” to “Participant characteristics” or “Sociodemographic characteristics”, as baseline implies that this was a prospective study. Also, the first sentence of this section only mentions sex, age, BMI and living arrangement but alcohol and smoking are also mentioned. In addition, please clarify if height and weight were measured by study staff or if BMI was calculated based on self-reported height and weight. Also, how close was the “index outpatient visit” to the time of the study measurements?

RESPONSE: Thank you for this comment. We have changed the subsection title from “Baseline characteristics” to “Participant characteristics” to better reflect the cross-sectional study design. We have also clarified the data-collection procedure. Upon arrival at the outpatient clinic, participants’ height and weight were measured by clinic nurses as part of the routine assessment and were not self-reported; BMI was subsequently calculated as weight in kilograms divided by height in meters squared. Patients were then invited to participate in the study, and those who provided informed consent underwent all remaining study assessments during the same outpatient visit. To avoid ambiguity, we replaced the term “index outpatient visit” with “study enrollment visit” and clarified that this visit occurred on the same day as the study assessments.

8. Data analysis: Please clarify why the prevalence of osteosarcopenia across comorbid conditions was assessed, as only general prevalence was mentioned in the study objective. In addition, the statement “Associations with osteosarcopenia…” is rather vague – I assume you mean associations between potential risk factors and prevalence of osteosarcopenia? Regarding the models with CVD – I don’t see results for two models presented. I’m also unclear why overall CVD would be included in a model with the individual components, as the composite variable wouldn’t carry any independent information. Please also clarify how regression diagnostics were checked.

RESPONSE: Thank you for these important comments. We retained the analysis of osteosarcopenia prevalence across comorbid conditions because these condition-specific estimates provide clinically relevant information and may highlight patient groups with a higher burden of osteosarcopenia who could benefit from more proactive screening and assessment. To align the study objective with the analyses performed, we revised the objective to explicitly include the estimation of both overall prevalence and prevalence across these selected conditions. We agree that the phrase “associations with osteosarcopenia” was insufficiently specific. Our intention was to examine the associations between potential explanatory factors and prevalent osteosarcopenia. Given the cross-sectional design, we reviewed the manuscript throughout and replaced causal terminology, including “risk factors,” with non-causal wording such as “associated factors” and “factors associated with prevalent osteosarcopenia.”

Our initial rationale for evaluating overall cardiovascular disease (CVD) was to combine participants with individual cardiovascular conditions and thereby improve statistical precision. However, after reconsidering this approach, we found that coronary artery disease (CAD) accounted for 96 of the 105 participants originally classified as having overall CVD. Therefore, the composite CVD variable provided little additional information beyond CAD and substantially overlapped with its individual component. We consequently removed overall CVD from all descriptive and regression analyses, deleted the previous description of two CVD models, and retained CAD as the cardiovascular variable in the primary multivariable model. Congestive heart failure and stroke, with only six and five cases, respectively, were retained in the exploratory univariable analyses but were not included in the primary conventional multivariable model because of their sparse cell counts. These conditions were subsequently evaluated in the Firth penalized multivariable logistic regression sensitivity analysis.

We also clarified the regression diagnostic procedures. All variance inflation factors were below 1.05, indicating no meaningful multicollinearity. The Hosmer–Lemeshow test showed no evidence of poor model fit (p = 0.109), and no observation had a Cook’s distance greater than 1, suggesting that no individual observation exerted undue influence on the model estimates. The functional forms of the continuous predictors were examined using the Box–Tidwell approach and a spline-based sensitivity analysis. BMI showed no evidence of departure from linearity, whereas a possible nonlinear association between age and prevalent osteosarcopenia was observed. Nevertheless, the spline-based sensitivity analysis yielded similar findings for the principal associated factors and did not alter the main conclusions. These revisions and diagnostic procedures have been clarified in the Data Analysis section.

9. Results: The term “rates” implies a time dimension – please remove this term from the results and discussion. P-values that are presented in the tables do not need to be repeated in the text.

RESPONSE: Thank you for this comment. We have removed the term “rates” and used “prevalence” consistently throughout the Results and Discussion, as appropriate for the cross-sectional study design. We have also removed p-values from the narrative Results where they were already presented in the corresponding tables or figure, while retaining the principal findings and effect estimates with 95% confidence intervals where informative.

10. Discussion: Since osteosarcopenia is spelled out in full elsewhere in the manuscript, I would suggest not using the abbreviation in the discussion.

RESPONSE: Thank you for this suggestion. We have replaced all instances of the abbreviation “OS” with the full term “osteosarcopenia” throughout the Discussion.

11. Discussion, limitations: Consider adding physical activity to the list of outcomes not assessed in this study, but which might provide further insight into mechanisms underpinning osteosarcopenia.

RESPONSE: Thank you for this suggestion. We have added physical activity to the limitations, together with other relevant factors that were not assessed. We have also noted that evaluating physical activity may provide further insight into the mechanisms underlying osteosarcopenia.

# REVIEWER 2

You have not mentioned the subjects' nutritional status, calcium uptake, and vitamin D status, which are relevant to the evaluation of osteosarcopenia. Have you used the physical activity (PA) questionnaire to assess PA?

RESPONSE: Thank you for this important comment. Detailed nutritional status beyond BMI, dietary calcium intake, and vitamin D status were not assessed in this study. In addition, a validated physical activity questionnaire was not administered. We have acknowledged the absence of these potentially relevant factors in the Limitations and noted that they should be considered in future studies.

Attachment Submitted filename: Response to Reviewers-16th Sept.docx

# Editor report: Decision Letter 2

**DOI:** 10.1371/journal.pone.0359850.r005

**Authors:** Heather Macdonald

20 Sep 2026

<p>Prevalence of Osteosarcopenia and Associated Factors Among Older Vietnamese Outpatients: A Cross-Sectional Study

PONE-D-26-13624R2

Dear Dr. Nguyen,

We’re pleased to inform you that your manuscript has been judged scientifically suitable for publication and will be formally accepted for publication once it meets all outstanding technical requirements.

Within one week, you’ll receive an e-mail detailing the required amendments. When these have been addressed, you’ll receive a formal acceptance letter and your manuscript will be scheduled for publication.

An invoice will be generated when your article is formally accepted. Please note, if your institution has a publishing partnership with PLOS and your article meets the relevant criteria, all or part of your publication costs will be covered. Please make sure your user information is up-to-date by logging into Editorial Manager at Editorial Manager® and clicking the ‘Update My Information' link at the top of the page. For questions related to billing, please contact billing support.

If your institution or institutions have a press office, please notify them about your upcoming paper to help maximize its impact. If they’ll be preparing press materials, please inform our press team as soon as possible -- no later than 48 hours after receiving the formal acceptance. Your manuscript will remain under strict press embargo until 2 pm Eastern Time on the date of publication. For more information, please contact onepress@plos.org.

Kind regards,

Heather Macdonald, Ph.D

Academic Editor

PLOS One

Additional Editor Comments (optional):

Thank you for addressing my comments - I don't have any additional concerns with the manuscript. I do note, however, that some of the references still need to be formatted correctly as not all journals are abbreviated (e.g., #13 & #29) and #27 requires a DOI.

Reviewers' comments:

# Editor report: Acceptance letter

**DOI:** 10.1371/journal.pone.0359850.r006

**Authors:** Heather Macdonald

PONE-D-26-13624R2

PLOS One

Dear Dr. Nguyen,

I'm pleased to inform you that your manuscript has been deemed suitable for publication in PLOS One. Congratulations! Your manuscript is now being handed over to our production team.

At this stage, our production department will prepare your paper for publication. This includes ensuring the following:

* All references, tables, and figures are properly cited

* All relevant supporting information is included in the manuscript submission,

* There are no issues that prevent the paper from being properly typeset

You will receive further instructions from the production team, including instructions on how to review your proof when it is ready. Please keep in mind that we are working through a large volume of accepted articles, so please give us a few days to review your paper and let you know the next and final steps.

Lastly, if your institution or institutions have a press office, please let them know about your upcoming paper now to help maximize its impact. If they'll be preparing press materials, please inform our press team within the next 48 hours. Your manuscript will remain under strict press embargo until 2 pm Eastern Time on the date of publication. For more information, please contact onepress@plos.org.

You will receive an invoice from PLOS for your publication fee after your manuscript has reached the completed accept phase. If you receive an email requesting payment before acceptance or for any other service, this may be a phishing scheme. Learn how to identify phishing emails and protect your accounts at https://explore.plos.org/phishing.

If we can help with anything else, please email us at customercare@plos.org.

Thank you for submitting your work to PLOS One and supporting open access.

Kind regards,

PLOS One Editorial Office Staff

on behalf of

Dr. Heather Macdonald

Academic Editor

PLOS One
